# A Translation-Aborting Small Open Reading Frame in the Intergenic Region Promotes Translation of a Mg^2+^ Transporter in *Salmonella* Typhimurium

**DOI:** 10.1128/mBio.03376-20

**Published:** 2021-04-13

**Authors:** Eunna Choi, Yoontak Han, Shinae Park, Hyojeong Koo, Jung-Shin Lee, Eun-Jin Lee

**Affiliations:** a Department of Life Sciences, School of Life Sciences and Biotechnology, Korea University, Seoul, South Korea; b Department of Molecular Bioscience, College of Biomedical Science, Kangwon National University, Chuncheon, South Korea; National Cancer Institute

**Keywords:** uORF, translation-inhibitory stem-loop structure, intergenic region, ribosome destabilization

## Abstract

Bacterial mRNAs often harbor upstream open reading frames (uORFs) in the 5′ untranslated regions (UTRs). Translation of the uORF usually affects downstream gene expression at the levels of transcription and/or translation initiation. Unlike other uORFs mostly located in the 5′ UTR, we discovered an 8-amino-acid ORF, designated *mgtQ*, in the intergenic region between the *mgtC* virulence gene and the *mgtB* Mg^2+^ transporter gene in the *Salmonella mgtCBRU* operon. Translation of *mgtQ* promotes downstream *mgtB* Mg^2+^ transporter expression at the level of translation by releasing the ribosome-binding sequence of the *mgtB* gene that is sequestered in a translation-inhibitory stem-loop structure. Interestingly, *mgtQ* Asp2 and Glu5 codons that induce ribosome destabilization are required for *mgtQ*-mediated *mgtB* translation. Moreover, the *mgtQ* Asp and Glu codons-mediated *mgtB* translation is counteracted by the ribosomal subunit L31 that stabilizes ribosome. Substitution of the Asp2 and Glu5 codons in *mgtQ* decreases MgtB Mg^2+^ transporter production and thus attenuates *Salmonella* virulence in mice, likely by limiting Mg^2+^ acquisition during infection.

## Abstract (precis)

Translation initiation regions in mRNAs that include the ribosome-binding site (RBS) and the start codon are often sequestered within a secondary structure. Therefore, to initiate protein synthesis, the mRNA secondary structure must be unfolded to allow the RBS to be accessible to the ribosome.

## INTRODUCTION

In bacteria, genes within an operon are transcribed as a single polycistronic mRNA and translated individually by allowing the ribosome access to the ribosome-binding sequence of each gene. However, translation of each gene within the operon is not fully independent, depending on the length of the intergenic region between the neighboring genes or the presence of specific regulatory mechanisms. Translation of the neighboring genes is usually tightly coupled when two genes are close to each other or overlapping because ribosome would simply reinitiate translation at the start codon of the following gene (1). However, given that many genes are separated by intergenic sequences ranging from a few to several hundred nucleotides (2, 3), additional regulatory mechanisms are often required to coordinate synthesis of proteins within the operon, which include interactions with proteins or small RNAs, endonucleolytic cleavage within mRNA, and translation of short open reading frames (ORFs) (4–7). Here, we identify a short ORF that is located in the intergenic region, translation of which promotes translation of the distal gene, thereby coupling protein synthesis between neighboring genes within the operon.

**FIG 1:**
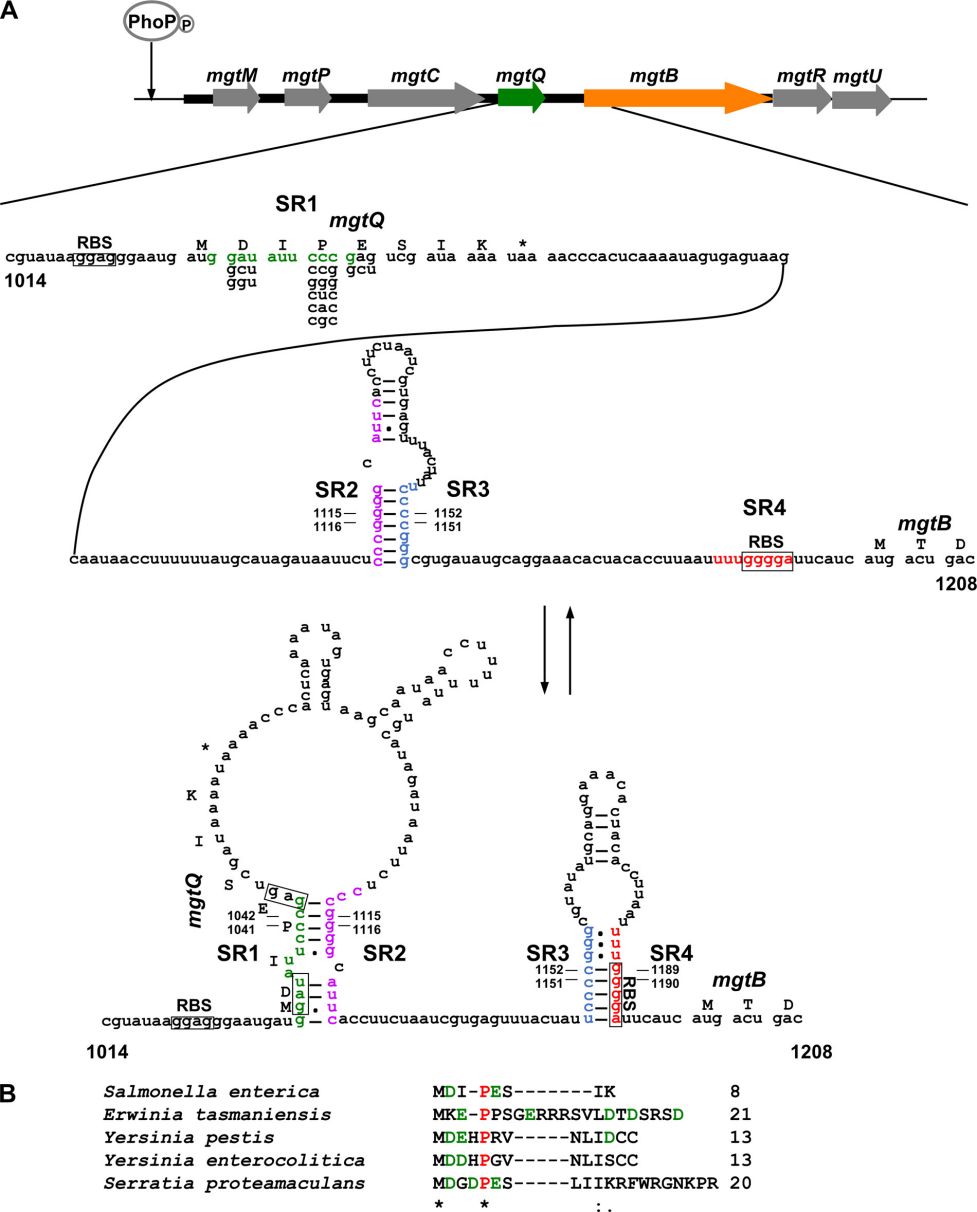
Regulation of the *Salmonella mgtCBRU* virulence operon by the *mgtQ* short ORF. (A) The phosphorylated PhoP response regulator binds to the *mgtCBRU* promoter and initiates transcription. Two short ORFs in the 5′ leader region, *mgtM* and *mgtP*, control transcription elongation in response to intracellular ATP and charged tRNA^Pro^ levels via transcription attenuation-like mechanisms. The 8-aa *mgtQ* ORF is located in the 219-nt intergenic region between *mgtC* and *mgtB* genes and harbors Asp and Glu codons at positions 2 and 5. The sequence overlapping the *mgtQ* ORF has the potential to adopt alternative RNA secondary structures (stem-loops 1:2 and 3:4 versus 2:3) and the *mgtB* RBS is occluded within the stem-loop 3:4 structure. The Asp and Glu codons at *mgtQ* appear to induce ribosome destabilization and promote the formation of stem-loop 2:3, thus releasing the RBS of the *mgtB* gene and enhancing *mgtB* translation. Positions of nucleotide substitutions used in the experiments presented in Fig. 4 are indicated. (B) Alignment of the deduced amino acid sequences of the *mgtQ* orthologs in the *mgtC-mgtB* intergenic regions from Salmonella enterica, Erwinia tasmaniensis, Yersinia pestis, Yersinia enterocolitica, and Serratia proteamaculans. Sequences in red correspond to Pro codons, and sequences in green correspond to Asp and Glu codons. Asterisks correspond to positions conserved in all listed species.

In the intracellular pathogen Salmonella enterica serovar Typhimurium, the *mgtB* gene encoding a Mg^2+^ transporting P-type ATPase lies between the virulence gene *mgtC* and the proteolysis-regulatory gene *mgtR* in the *mgtCBRU* operon. The *mgtCBRU* operon is transcribed from a single promoter located upstream of the first *mgtC* gene, and the MgtB Mg^2+^ transporter is produced from a part of >4-kb polycistronic mRNA (8). (Although there appeared to be a read-through transcript into the fifth gene *cigR*, we only focused on four genes here because the *cigR* gene is also transcribed from a constitutive promoter [9]). Because the MgtB protein imports Mg^2+^ ions at the expense of ATP (10), it is reasonable that the *mgtCBRU* operon is highly transcribed in low Mg^2+^ by the PhoP/PhoQ two-component system (11) to support bacterium’s growth in a Mg^2+^-limiting condition. However, although the *mgtCBRU* operon is transcribed as a single polycistronic message, the production of the MgtB Mg^2+^ transporter appears to be differentially regulated based on the following reasons. First, the first gene product, MgtC, is a virulence protein that inhibits F_o_F_1_ ATP synthase, thus decreasing ATP production (12). Given that one of the major biological functions of Mg^2+^ ions is neutralizing free ATP as well as other nucleotides (13, 14), the requirement of the MgtB protein importing Mg^2+^ ions may vary depending on the amount of the active MgtC protein in a given condition. Second, the intergenic region between the first *mgtC* and the second *mgtB* genes is 219 nucleotides (nt) long, which is relatively long considering the average length of the intergenic regions in bacteria (15, 16). Lastly, Mfold analysis predicted that the ribosome-binding site (RBS) of the *mgtB* gene is occluded by a potential base pairing with upstream sequences (Fig. 1) (17), suggesting that an additional regulatory element is required for *mgtB* translation.

In this study, we discovered a short ORF, designated *mgtQ*, in the intergenic region between the *mgtC* and *mgtB* genes. Translation of *mgtQ* is required for downstream *mgtB* translation. *Salmonella*’s intrinsic ability to translate *mgtQ* is dependent on acidic residues in *mgtQ*, which are known to induce ribosomal destabilization and also require a specific ribosomal subunit encoded by the *rpmE1* gene to counteract the acidic residue-mediated translation abortion. Therefore, translation efficiency of *mgtQ* is involved in controlling production of the MgtB Mg^2+^ transporter, thus contributing to the ability to import Mg^2+^ and *Salmonella* pathogenesis.

## RESULTS

### The intergenic region between the *mgtC* and *mgtB* genes includes a short ORF.

**FIG 2:**
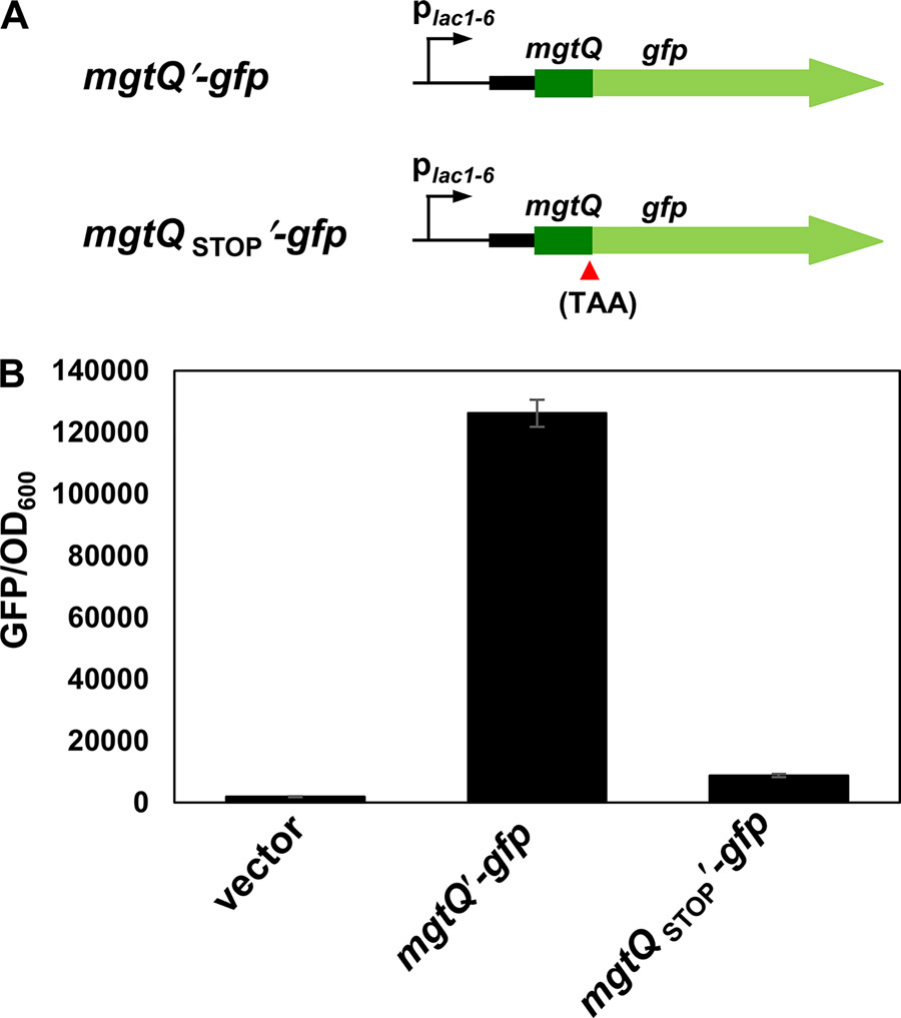
The *mgtQ* ORF is translated *in vivo*. (A) Schematic representation of *mgtQ*′-*gfp* constructs. (B) Fluorescence produced by wild-type *Salmonella* (14028s) harboring the plasmid vector (ptGFP), or derivatives with a *gfp* translational fusion to the last *mgtQ* codon (p*mgtQ′-gfp*), or following the stop *mgtQ* codon (p*mgtQ*_STOP_′-*gfp*). Bacteria were grown for 4 h in N-minimal medium containing 10 mM Mg^2+^ as described in Materials and Methods. The means and standard deviations (SD) from three independent measurements are shown.

The *mgtCBRU* operon is transcribed as a polycistronic mRNA from the single promoter located upstream of the *mgtC* gene (8). Interestingly, the *mgtC* and *mgtB* genes are separated by the 219-nt intergenic region, which is unusually long for the operon (15, 16). The presence of the long intergenic region led us to examine whether it contains an additional regulatory element. We found a potential short ORF located 51 nt downstream of the *mgtC* stop codon, designated *mgtQ*. *mgtQ* is an 8-amino acid ORF preceded by a strong Shine-Dalgarno RBS (AGGAGG) (Fig. 1; see also Fig. S1 in the supplemental material). Although it varies in length, the presence of *mgtQ* is conserved in other bacteria, including Erwinia tasmaniensis, Yersinia pestis, Yersinia enterocolitica, and Serratia proteamaculans (Fig. 1; see also Fig. S1 and S2). The conservation of the *mgtQ* ORF in other bacteria raises the possibility that the *mgtQ* ORF is translated. To test this, we constructed a strain harboring a promoterless *gfp* plasmid translationally fused to the DNA fragment corresponding to nt 982 to 1054 (from the intergenic region to the last sense codon of the *mgtQ* ORF). As a control, we used an isogenic derivative in which the *gfp* gene was fused after the *mgtQ* stop codon (*mgtQ*_STOP_′-*gfp*) (Fig. 2A). The strain harboring *mgtQ′-gfp* produced high levels of fluorescence compared to control strains with the empty vector or *mgtQ*_STOP_′-*gfp* (Fig. 2B). This result indicates that *mgtQ* is translated *in vivo*.

10.1128/mBio.03376-20.2 FIG S1 The intergenic regions between the *mgtC* and *mgtB* genes of other enteric bacteria harbor *mgtQ* orthologs. Alignment of the nucleotide sequences corresponding to part of the intergenic regions between the *mgtC* and *mgtB* genes from Salmonella enterica, Erwinia tasmaniensis, Yersinia pestis, Yersinia enterocolitica, and Serratia proteamaculans. Sequences in boldface correspond to *mgtQ*. Sequences in green, purple, blue, and red represent regions involved in stem-loop structures (SR1, SR2, SR3, and SR4, respectively) shown in Fig. 1. The predicted RBS for each ORF is underlined. Asterisks correspond to nucleotides conserved in all listed species. Download FIG S1, TIF file, 0.2 MB. Copyright © 2021 Choi et al. 2021 Choi et al. https://creativecommons.org/licenses/by/4.0/ This content is distributed under the terms of the Creative Commons Attribution 4.0 International license.

10.1128/mBio.03376-20.3 FIG S2 The presence of *mgtQ* is conserved in enteric bacteria where the *mgtC* and *mgtB* genes are in the same operon. In Salmonella enterica, *mgtQ* is located in the *mgtC-mgtB* intergenic region. Similarly, the *mgtC-mgtB* intergenic regions from Erwinia tasmaniensis, Yersinia pestis, Yersinia enterocolitica, and Serratia proteamaculans include *mgtQ*-like ORFs. In Brucella melitensis, the *mgtC* and *mgtB* genes appear to be part of separate transcription units. An *mgtQ*-like sequence is found upstream of the *mgtB* gene. Mycobacterium tuberculosis harbors the *mgtC* gene but not *mgtB* or *mgtQ*. Download FIG S2, TIF file, 0.2 MB. Copyright © 2021 Choi et al. 2021 Choi et al. https://creativecommons.org/licenses/by/4.0/ This content is distributed under the terms of the Creative Commons Attribution 4.0 International license.

### *mgtQ* affects *mgtB* expression at the level of translation.

**FIG 3:**
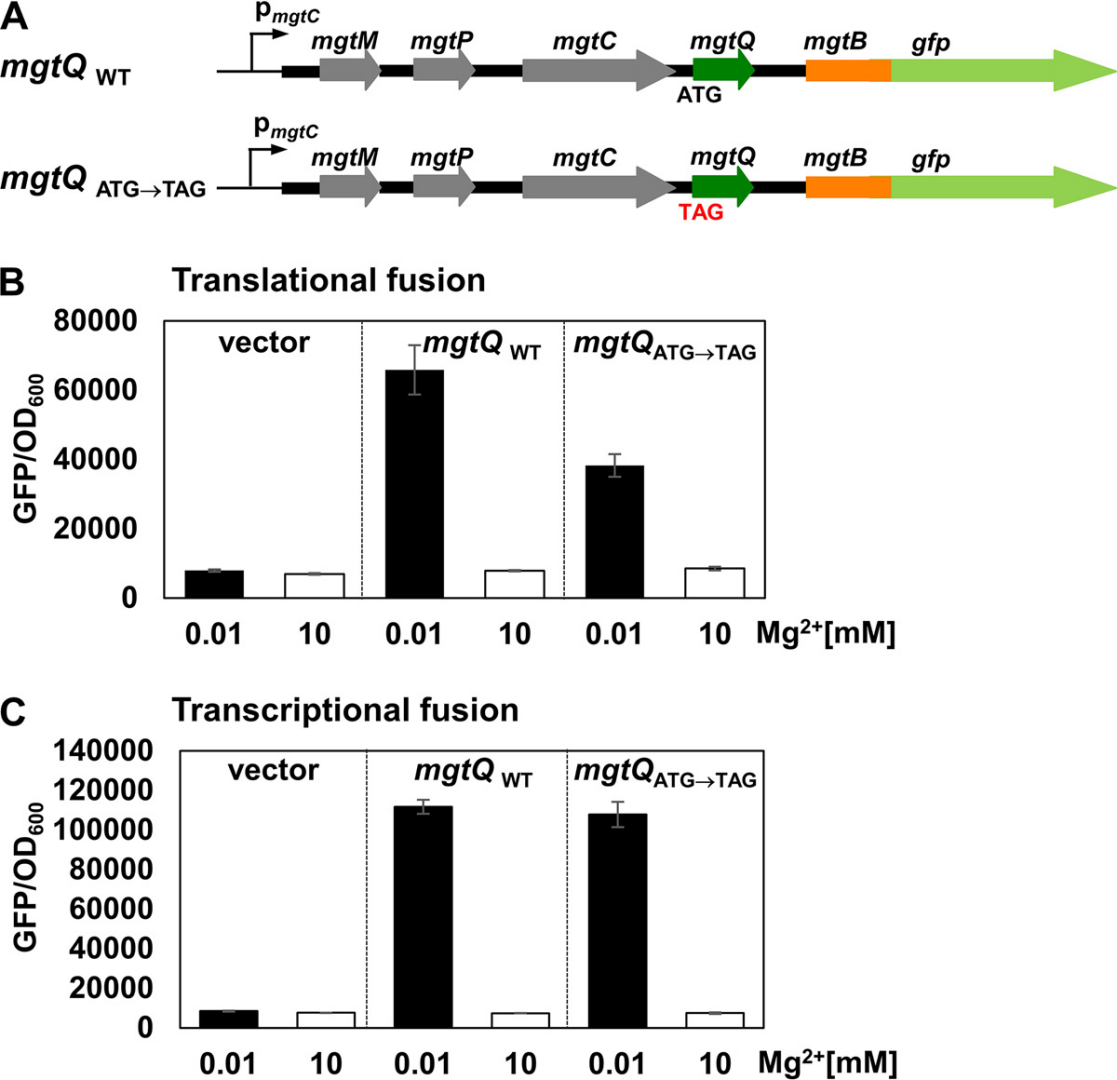
*mgtQ* translation affects *mgtB* expression at a translational level but not transcriptional level. (A) Schematic representation of *mgtB-gfp* constructs used in this experiment. (B and C) Fluorescence produced by wild-type *Salmonella* (14028s) harboring a plasmid with either a *gfp* gene translationally (B) or transcriptionally (C) fused to the *mgtB* gene with the wild-type *mgtQ* (*mgtQ*_WT_) or an *mgtQ* mutant with the start codon substituted by the TAG stop codon (*mgtQ*_ATG→TAG_). Bacteria were grown for 5 h in N-minimal medium containing 0.01 mM (inducing) or 10 mM (noninducing) Mg^2+^ as described in Materials and Methods. The means and SD from three independent measurements are shown.

Because *mgtQ* is located upstream of the *mgtB* gene (Fig. 1), we hypothesized that *mgtQ* impacts downstream *mgtB* expression. To test *mgtQ*’s effect on *mgtB* expression, we constructed a plasmid with the *gfp* gene fused transcriptionally or translationally to the first 27 codons of the *mgtB* gene (Fig. 3A; see also Fig. S3). We also constructed an isogenic plasmid harboring the *mgtB*-fused *gfp* gene with the upstream *mgtQ* start codon substituted by the TAG stop codon (*mgtQ*_ATG→TAG_; Fig. 3A). Because both plasmids harbor a 1.2-kb DNA fragment containing the PhoP-dependent promoter (p*_mgtC_*), as well as the *mgtC* and *mgtQ* genes upstream of the *mgtB-*fused *gfp* gene, we grew *Salmonella* strains harboring the *mgtB-*fused *gfp* plasmids in PhoP-inducing (0.01 mM Mg^2+^) or PhoP-repressing (10 mM Mg^2+^) conditions. A *Salmonella* strain harboring the plasmid with the wild-type *mgtQ* produced high levels of *mgtB-gfp* fluorescence in 0.01 mM Mg^2+^, but low levels of fluorescence in 10 mM Mg^2+^ for both transcriptional and translational fusions (Fig. 3B and C). However, the introduction of the start to stop codon at *mgtQ* decreased *mgtB-gfp* fluorescence when fused translationally but not when fused transcriptionally (Fig. 3B and C). This indicates that *mgtQ* affects *mgtB* expression at translational level. As controls, strains harboring the empty vector or strains grown in the noninducing media exhibited low levels of fluorescence in all tested conditions (Fig. 3B and C).

10.1128/mBio.03376-20.4 FIG S3 Schematic representation of reporter systems used in this study. (A) A partial sequence of pfpv25 plasmid, a plasmid for transcriptional fusion to a promoterless *gfp* gene. (B) A partial sequence of ptGFP, an engineered plasmid for translational fusion to a promoterless *gfp* gene. In ptGFP plasmid, a portion of sequence in pfpv25 (indicated in blue) was deleted to remove the ribosome binding site and the start codon of the *gfp* gene. Download FIG S3, TIF file, 0.09 MB. Copyright © 2021 Choi et al. 2021 Choi et al. https://creativecommons.org/licenses/by/4.0/ This content is distributed under the terms of the Creative Commons Attribution 4.0 International license.

We also created a chromosomal mutant with the same start to stop codon substitution in *mgtQ* (see Fig. S4). Similarly to what we observed in *gfp* plasmids, the introduction of the TAG stop codon at *mgtQ* severely decreased MgtB protein levels in the PhoP-inducing condition, while the same substitution did not affect mRNA levels of the *mgtC* and *mgtB* genes (see Fig. S4). These results further demonstrate that *mgtQ* indeed affects *mgtB* expression at the translational level.

10.1128/mBio.03376-20.5 FIG S4 *mgtQ* translation increases MgtB protein levels but not mRNA levels. (A) Schematic representation of an *mgtQ* chromosomal mutant with the start codon substituted by the stop codon and the DNA fragments amplified by QRT-PCR in panels D and E. (B and C) Western blot analysis of crude extracts prepared from *Salmonella* strains with the wild-type *mgtQ* (*mgtQ*_WT_) or the *mgtQ* derivative with the start to stop codon substitution (*mgtQ*_ATG→TAG_) grown for 5 h in N-minimal media containing 10 or 0.01 mM Mg^2+^. The amounts of MgtB and Fur proteins were determined by anti-MgtB (B) and anti-Fur (C) antibodies. (D and E) Relative mRNA levels of the *mgtC* (D) and *mgtB* (E) coding regions produced by wild-type (*mgtQ*_WT_) *Salmonella* and the *mgtQ* mutant with the start codon replaced by the stop codon (*mgtQ*_ATG→TAG_) grown for 5 h in N-minimal media containing 10 or 0.01 mM Mg^2+^. The means and SD from three independent measurements are shown. Download FIG S4, TIF file, 0.1 MB. Copyright © 2021 Choi et al. 2021 Choi et al. https://creativecommons.org/licenses/by/4.0/ This content is distributed under the terms of the Creative Commons Attribution 4.0 International license.

### Formation of alternative stem-loop structures overlapping the *mgtQ* ORF is involved in controlling *mgtB* translation.

**FIG 4:**
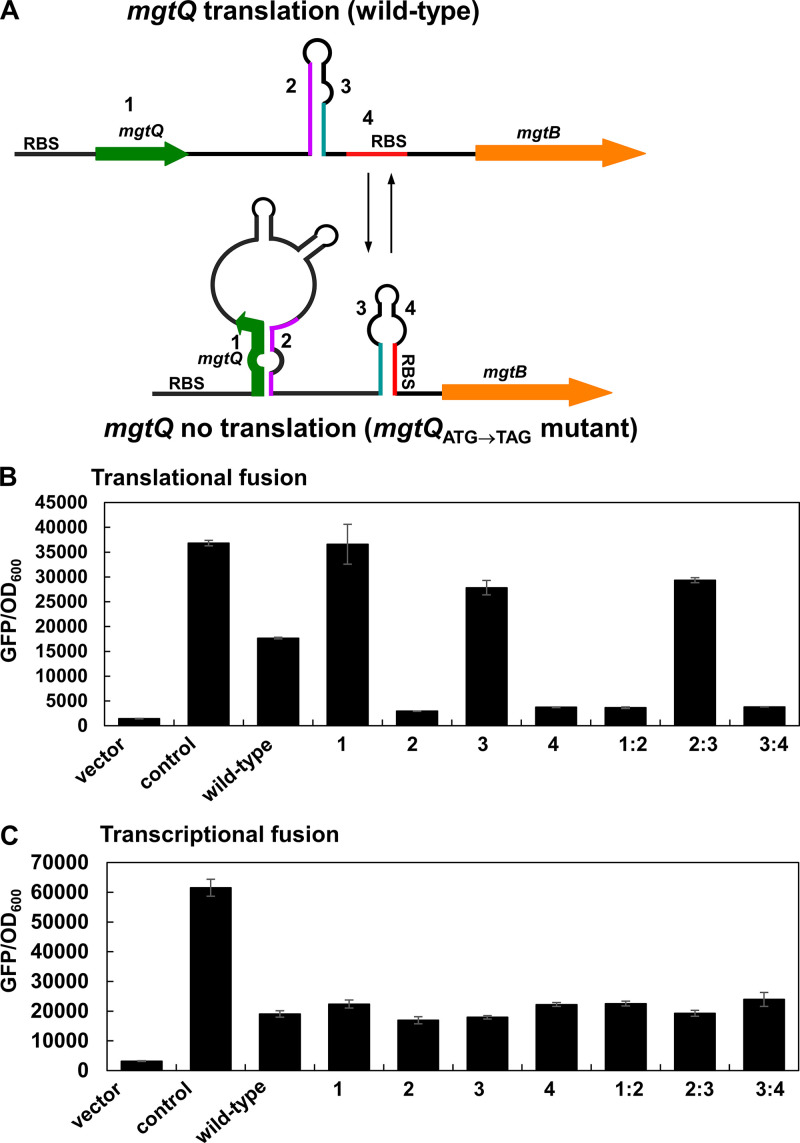
Formation of alternative stem-loop structures that include *mgtQ* affects downstream *mgtB* translation. (A) Schematic representation of a model showing predicted alternative stem-loop structures (1:2 and 3:4 versus 2:3) that are associated with the *mgtQ* ORF. (B) Fluorescence produced by wild-type *Salmonella* (14028s) harboring the plasmid vector (vector), or derivatives with a *gfp* translational fusion to the *mgtB* gene that includes the upstream intergenic region (wild-type), substitution mutations in the stem regions 1, 2, 3, or 4 that hinder stem-loop formation (1:2, 2:3, and/or 3:4), or compensatory mutations that recover the formation of stem-loops 1:2, 2:3, or 3:4. A strain harboring a plasmid with a *gfp* translational fusion derived from the p*_lac_*_1-6_ promoter was used as a positive control. Bacteria were grown for 4 h in N-minimal media containing 0.01 mM Mg^2+^ as described in Materials and Methods. The means and SD from three independent measurements are shown. (C) Fluorescence produced by wild-type *Salmonella* (14028s) harboring the plasmid vector (vector), or derivatives with a *gfp* transcriptional fusion to the *mgtB* gene that includes the upstream intergenic region (wild-type), substitution mutations described in panel B. A strain harboring a plasmid with a *gfp* transcriptional fusion derived from the p*_lac_*_1-6_ promoter was used as a positive control. Bacteria were grown for 4 h in N-minimal media containing 0.01 mM Mg^2+^ as described in Materials and Methods. The means and SD from three independent measurements are shown.

We then wondered how *mgtQ* affects downstream *mgtB* translation. Sequence analyses of the *mgtC-mgtB* intergenic region revealed that the sequence overlapping with the *mgtQ* ORF has the potential to adopt two alternative stem-loop structures: stem-loops 1:2 and 3:4 or stem-loop 2:3 (Fig. 1 and Fig. 4A). The presence of alternative stem-loop structures neighboring the *mgtQ* ORF suggests that *mgtQ* translation might affect the formation of one of two alternative stem-loop structures, which eventually leads to an increase or decrease in downstream *mgtB* expression. Interestingly, among those stem-loop structures, stem-loop 3:4 appears to be a translation-inhibitory stem-loop structure because the formation of stem-loop 3:4 occludes the RBS of the *mgtB* gene (Fig. 4A). Given that the formations of stem-loop 2:3 and stem-loops 1:2/3:4 are mutually exclusive, the formation of stem-loop 2:3 is expected to increase *mgtB* translation by preventing the formation of stem-loop 3:4 and liberating the RBS of the *mgtB* gene (Fig. 4B). To explore this, we introduced substitutions in stem regions 1, 2, 3, or 4 of the *mgtB*-fused *gfp* plasmids to determine the effect of stem-loop formation on downstream *mgtB* expression. A nucleotide substitution in stem region 3 that is expected to hinder the formation of both stem-loop 3:4 and stem-loop 2:3 increased translationally fused *mgtB-gfp* expression (*mgtB*′-*gfp*), supporting the idea that stem-loop 3:4 is a translation-inhibitory stem-loop structure (Fig. 4B).

In contrast, the substitution of stem region 4 decreased *mgtB*′-*gfp* expression due to loss of the RBS of the *mgtB* gene (Fig. 4B). Substitution in stem region 2 that appears to disrupt the formation of stem-loop 2:3 and thus favor the formation of stem-loop 3:4, resulted in a decrease in *mgtB*′-*gfp* levels (Fig. 4B). Similarly, substitution in stem region 1 within *mgtQ* increased *mgtB*′-*gfp* levels because the substitution disrupts the formation of stem-loop 1:2 and favors the formation of stem-loop 2:3, hence releasing the RBS of the *mgtB* gene (Fig. 4B). The introduction of a compensatory mutation in either stem-loop 1:2 or 3:4 is expected to force the formation of stem-loop 3:4 and thus decreased *mgtB*′-*gfp* expression (Fig. 4B). In contrast, the introduction of a compensatory mutation in stem-loop 2:3 that possibly fails to occlude the RBS of the *mgtB* gene increased *mgtB*′-*gfp* expression. All substitutions in the stem-loop regions described above affect *mgtB*′-*gfp* expression only at translational level because isogenic substitutions did not have an effect on *mgtB-gfp* expression when fused transcriptionally (Fig. 4C). These data demonstrate that two alternative stem-loop structures could be formed and control *mgtB* translation by either masking or revealing the RBS of the downstream *mgtB* gene.

### Stem-loop 1:2 formation but not *mgtQ* Pro codon is required for *mgtB* translation.

As described above, inhibition of *mgtQ* translation by introducing the *mgtQ* start codon mutation decreased *mgtB* translation, likely because stem-loops 1:2 and 3:4 are formed when *mgtQ* is not translated (Fig. 4A). Moreover, the substitution in stem region 1 within *mgtQ* increased *mgtB* translation by releasing the RBS from the inhibitory stem-loop structure 3:4. Because stem-loop 1:2 is also disrupted while *mgtQ* is translated, any condition that slows down *mgtQ* translation is expected to promote *mgtB* translation in *cis* by favoring the formation of stem-loop 2:3 (Fig. 4A).

**FIG 5:**
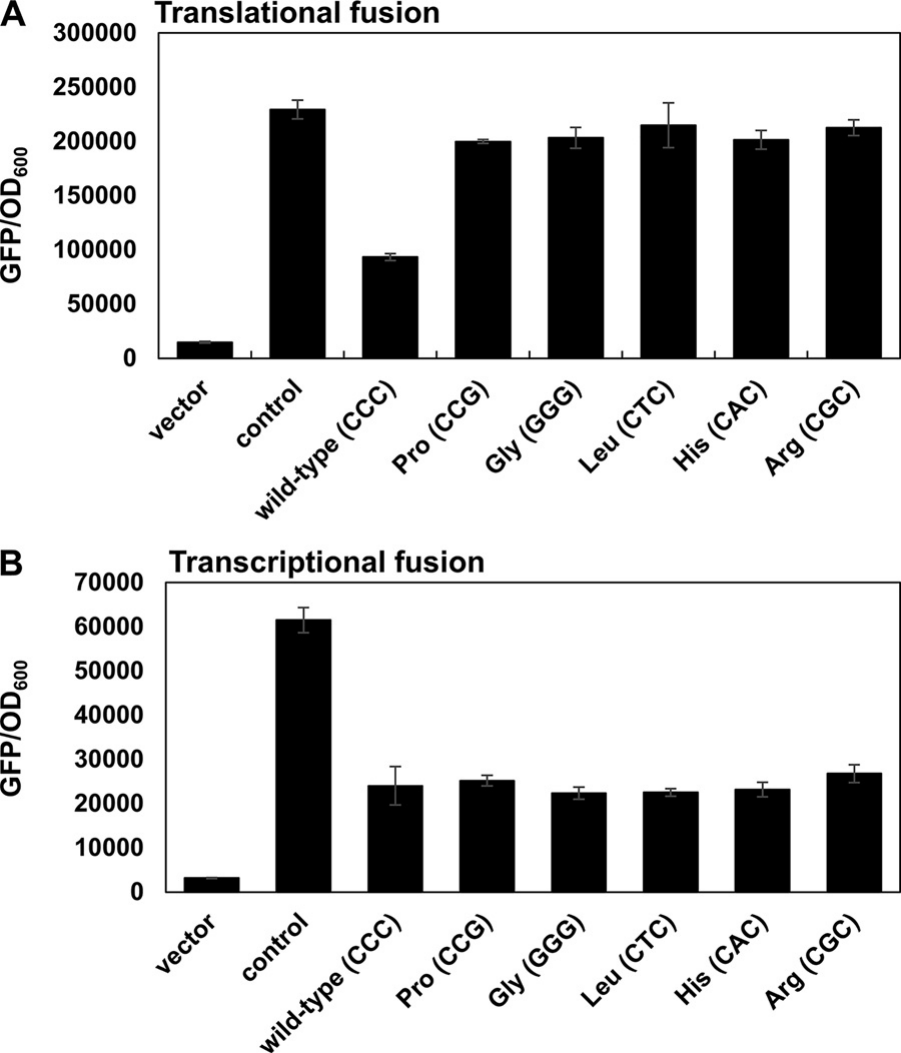
Stem-loop formation but not *mgtQ* Pro codon is required for *mgtQ*-mediated *mgtB* translation. (A and B) Fluorescence produced by wild-type *Salmonella* (14028s) harboring the plasmid vector (vector), or derivatives with a *gfp* translational (A) or transcriptional (B) fusion to the *mgtB* gene that includes the wild-type *mgtQ*, nucleotide substitution mutations of the conserved proline codon replaced by a synonymous mutation (CCG) or nonsynonymous mutations, including Gly (GGG), Leu (CTC), His (CAC), and Arg (CGC). The strains harboring a plasmid with a *gfp* translational fusion derived from the p*_lac_*_1-6_ promoter were used as positive controls. Bacteria were grown for 5 h in N-minimal media containing 0.01 mM Mg^2+^ as described in Materials and Methods. The means and SD from three independent measurements are shown.

Given that the *mgtQ* ORF has a single conserved proline codon at the fourth position (Fig. 1B) and that the nucleotide sequence of the proline codon (CCC) is a part of the stem region 1 (Fig. 1), we wondered whether the conserved proline codon has a role to affect downstream *mgtB* translation. To test this, we substituted the Pro codon in *mgtQ* (CCC) with Gly (GGG), Leu (CTC), His (CAC), or Arg (CGC) codons and measured the downstream *mgtB*′-*gfp* levels. All of these *mgtQ* Pro substitutions to Gly, Leu, His, or Arg increased *mgtB*′-*gfp* levels compared to levels in the isogenic control with the wild-type *mgtQ* (Fig. 5A), suggesting that removal of the *mgtQ* Pro codon might have an effect on downstream *mgtB* expression. However, we found that the silent substitution of the Pro codon CCC to CCG still increased downstream *mgtB*′-*gfp* levels similar to those detected in other missense mutations (Fig. 5A). Thus, disruption of stem-loop 1:2 rather than removal of the Pro codon determines *mgtB*′-*gfp* expression by promoting the release of the *mgtB* RBS. It is interesting to note that, although the substitution of the Pro codon (CCC) with Leu codon (CTC) was expected to form stem-loop 1:2 by non-Watson Crick base-pairing (U:G), it seemed to fail to form stem-loop 1:2, because the substitution still increased *mgtB′-gfp* levels (Fig. 5A). As a control experiment, isogenic substitutions in transcriptionally fused *mgtB-gfp* plasmids did not have any effect on *mgtB* expression (Fig. 5B).

### Ribosome-destabilizing acidic residues in *mgtQ* are required for *mgtB* translation.

During the multiple rounds of translation, if the *mgtQ*-translating ribosome continues to occupy stem region 1, the transcribed mRNAs thermodynamically favor the formation of stem-loop 2:3 over the formation of stem-loop 3:4 (the former free energy is −16.4 kcal/mol and the latter is −12.26 kcal/mol), thereby increasing *mgtB* translation. The fact that the *mgtQ* start codon mutation decreased *mgtB* translation (Fig. 3; see also Fig. S4) suggests that the *mgtQ*-translating ribosome might be a determinant to disrupt stem-loop 1:2 formation, allowing the formation of stem-loop 2:3 and increasing *mgtB* expression. In addition, the *mgtQ* substitution that does not affect codons but disrupts stem-loop 1:2 formation exhibited higher levels of *mgtB′-gfp* than those with the wild-type *mgtQ* sequence (Fig. 5), suggesting that completion of *mgtQ* translation appears to provide a chance to reassociate stem-loop 1:2 and thus decreases *mgtB* translation unless stem-loop 1:2 formation is disrupted by the nucleotide substitutions.

**FIG 6:**
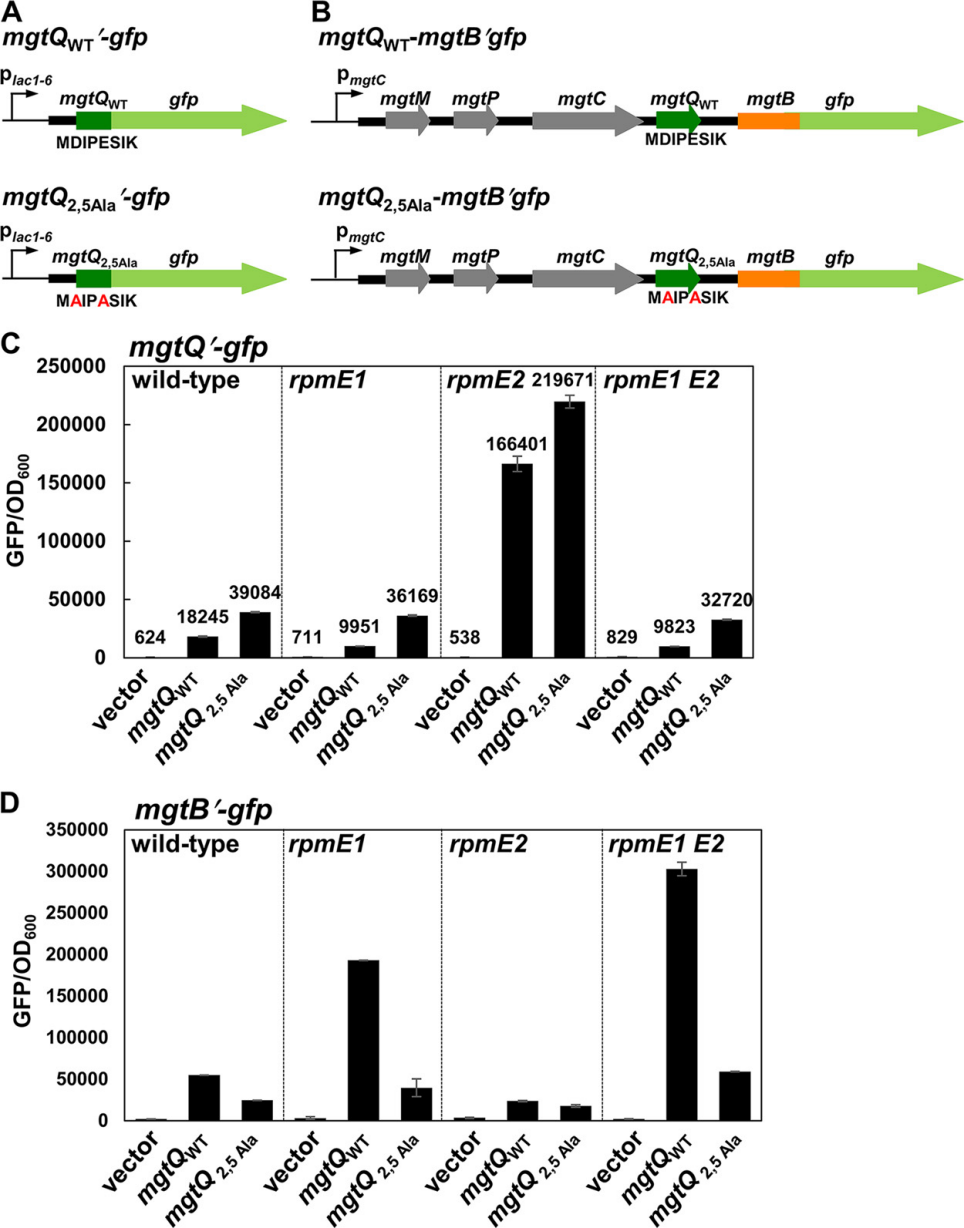
Asp2 and Glu5 codons in *mgtQ* are required for *mgtB* translation. (A) Schematic representation of *mgtQ′-gfp* constructs used in this experiment. (B) Schematic representation of *mgtB′-gfp* constructs used in this experiment. (C) Fluorescence produced by wild-type *Salmonella* (14028s) by *rpmE1* (EN1119), *rpmE2* (EN1120), or *rpmE1 rpmE2* (EN1368) *Salmonella* isolates harboring the promoterless plasmid vector (ptGFP), or by derivatives with *gfp* translational fusions to the wild-type *mgtQ* (p*mgtQ′-gfp*) or Asp2, Glu5 to Ala-substituted *mgtQ* (p*mgtQ*_2,5Ala_′-*gfp*). (D) Fluorescence produced by wild-type *Salmonella* (14028s), by *rpmE1* (EN1119), *rpmE2* (EN1120), or *rpmE1 rpmE2* double mutant (EN1368) *Salmonella* harboring the plasmid vector (vector), or by *Salmonella* derivatives with a *gfp* translational fusion to the *mgtB* gene that includes the wild-type *mgtQ* or nucleotide substitution mutation of the Asp2 and Glu5 codons replaced with Ala. Bacteria were grown for 4 h in N-minimal media containing 0.01 mM Mg^2+^ as described in Materials and Methods. The means and SD from three independent measurements are shown.

Although the *mgtQ* Pro4 codon affected *mgtB* translation by participating in base pairing of stem-loop 1:2 (Fig. 5), Pro4 might not affect *mgtQ* translation because single proline codon is not known to affect ribosome stalling. We then wondered what other factors could affect *mgtQ* translation. In Escherichia coli, acidic residues such as Asp and Glu in the nascent peptide chain interact within the ribosome exit tunnel and destabilize the translating ribosome during elongation (18). Because the *mgtQ* sequence also contains Asp and Glu codons at positions 2 and 5, respectively, we investigated whether these residues affect *mgtQ* translation by introducing Ala codon substitutions (Fig. 6A). A strain harboring the *mgtQ′-gfp* plasmid with Asp2 and Glu5 codons substituted by Ala codons exhibited a 2.1-fold increase in fluorescence compared to those produced from *mgtQ*_WT_*′-gfp* (Fig. 6C), indicating that Asp and Glu codons indeed decrease *mgtQ* translation. To detect *mgtQ* translation from its chromosomal location, we created chromosomal mutants with a C-terminal 8×myc tag fused to the *mgtQ*_WT_ or *mgtQ*_2,5Ala_ genes (see Fig. S5). MgtQ_WT_-8×myc fusion proteins were marginally detectable in the PhoP-inducing condition (see Fig. S5). However, when we substituted the Asp2 and Glu5 to Ala codons, MgtQ_2,5Ala_-8×myc fusion protein production was strongly enhanced in the same condition (see Fig. S5), indicating that the presence of the *mgtQ* Asp2 and Glu5 residues impedes *mgtQ* translation. This is also in agreement with the previous finding that acidic residues destabilize the translating ribosome (18), although it has not been demonstrated here.

10.1128/mBio.03376-20.6 FIG S5 The Asp2 and Glu5 to Ala substitutions enhance detection of the C-terminally 8×myc-tagged MgtQ peptides. (A) Schematic representation of *Salmonella* strains used in this study. (B-C) Western blot analysis of crude extracts prepared from *Salmonella* strains with the C-terminally 8×myc-tag fused to the wild-type *mgtQ* (EN1469) or *mgtQ*_2,5Ala_ (EN1471) genes probed with anti-Myc (B) or anti-Fur (C) antibodies to detect MgtQ or Fur proteins, respectively. MgtQ-8×myc showed a higher SDS-PAGE-displayed molecular weight (∼20 kDa) than its predicted size (10.4 kDa) due to high content of acidic amino acids (47.7% in MgtQ_WT_-8×myc and 45.5% in MgtQ_2,5Ala_-8×myc) (Y. Guan, Q. Zhu, D. Huang, S. Zhao, L. Jan Lo, J. Peng, Sci Rep 5:13370, 2015, doi:10.1038/srep13370). Bacteria were grown for 5 h in N-minimal media containing 10 or 0.01 mM Mg^2+^ as described in Materials and Methods. Download FIG S5, TIF file, 0.1 MB. Copyright © 2021 Choi et al. 2021 Choi et al. https://creativecommons.org/licenses/by/4.0/ This content is distributed under the terms of the Creative Commons Attribution 4.0 International license.

We then tested the effect of the *mgtQ* Asp2 and Glu5 to Ala substitutions on *mgtB* translation by introducing the same *mgtQ* substitution to a plasmid harboring *mgtB′-gfp* expressed from the native p*_mgtC_* promoter (Fig. 6B). The *mgtQ*_2,5Ala_ substitution decreased *mgtB′-gfp* levels compared to those with *mgtQ*_WT_ (Fig. 6D), while the same substitution increased *mgtQ′-gfp* levels (Fig. 6C). This demonstrates that *mgtQ* translation efficiency inversely correlates with that of the *mgtB* gene. Given that Asp2 and Glu5 codons decrease *mgtQ* expression, *mgtQ* Asp2 and Glu5 codons appear to induce ribosome destabilization at *mgtQ*, favoring the formation of stem-loop 2:3 and releasing the RBS of the *mgtB* gene for promoting translation.

One can argue that the *mgtQ* Asp2 and Glu5 to Ala (GCT) substitutions might affect mRNA secondary structure formation and thus *mgtB* expression. To exclude this possibility, we created a similar derivative of *mgtQ′-gfp* with Asp2 and Glu5 substituted by Gly2 (GGT) and Ala5 (GCT) codons that potentially maintain base-pairing between stem regions 1 and 2 (see Fig. S6). The Asp2-to-Gly (GGT) and Glu5-to-Ala (GCT) substitutions increased the *mgtQ′-gfp* levels, similar to those detected in the *mgtQ* Asp2 and Glu5 to Ala (GCT) substitutions. Moreover, when we introduced the same *mgtQ* substitution into the *mgtB′-gfp* plasmid, the Asp2-to-Gly (GGT) and Glu5-to-Ala (GCT) substitutions inversely decreased *mgtB′-gfp* levels (see Fig. S6), further supporting that acidic residues in *mgtQ* reduce *mgtQ* translation, thus increasing downstream *mgtB* expression at translational level.

10.1128/mBio.03376-20.7 FIG S6 Asp2 and Glu5 codons in *mgtQ* are required for *mgtB* translation independently of the ability to form stem-loop 1:2. (A) A possible secondary structure of *mgtQ*-harboring mRNA sequences. Substituted nucleotide sequences of Asp2 and Glu5 codons in *mgtQ* are indicated at the side of Asp2 and Glu5 sequences. (B-C) Schematic representation of *mgtQ′-gfp* (B) and *mgtB′-gfp* constructs (C) used in this experiment. (D) Fluorescence produced by wild-type (14028s) *Salmonella* harboring the promoterless plasmid vector (ptGFP), or derivatives with a *gfp* translational fusion to the wild-type *mgtQ* (p*mgtQ′-gfp*) or Asp2 (GAT) Glu5 (GAG) to Gly2 (GGT) Ala5 (GCT)-substituted *mgtQ* (p*mgtQ*_2Gly, 5Ala_′-*gfp*). (E) Fluorescence produced by wild-type (14028s) *Salmonella* harboring the plasmid vector (vector), or derivatives with a *gfp* translational fusion to the *mgtB* gene that includes the wild-type *mgtQ* or the *mgtQ* mutant with the Asp2 and Glu5 substitutions as described previously (D). Bacteria were grown for 4 h in N-minimal media containing 0.01 mM Mg^2+^ as described in Materials and Methods. Means and SD from three independent measurements are shown. Download FIG S6, TIF file, 0.1 MB. Copyright © 2021 Choi et al. 2021 Choi et al. https://creativecommons.org/licenses/by/4.0/ This content is distributed under the terms of the Creative Commons Attribution 4.0 International license.

### L31 ribosomal protein encoded by the *rpmE1* gene controls *mgtQ* translation via acidic residues.

Chadani et al. (18) previously reported that E. coli L31 intersubunit bridge counteracts ribosome destabilization mediated by acidic residues in the nascent peptide. Because Salmonella enterica contains two types of L31 proteins encoded by *rpmE1* and *rpmE2* genes, respectively, we constructed chromosomal mutant strains deleted the *rpmE1* and/or *rpmE2* genes. The *rpmE1* mutant exhibited decreased *mgtQ′-gfp* levels, and thus further increased *mgtB′-gfp* levels (Fig. 6C and D). Alterations of *mgtQ*′-*gfp* and *mgtB*′-*gfp* levels in the *rpmE1* mutant were Asp2 and Glu5 dependent because the *rpmE1* mutation had no further effect on Asp2- and Glu5-substituted *mgtQ′-gfp* levels or *mgtQ*_2,5Ala_-containing *mgtB′-gfp* levels (Fig. 6C and D). These data suggest that the L31 ribosomal protein encoded by the *rpmE1* gene appears to be involved in resolving the acidic residue-mediated ribosomal destabilization at *mgtQ*, thereby decreasing *mgtB* translation. Interestingly, the *rpmE2* mutation further increased *mgtQ′-gfp* levels, implicating that the L31 ribosomal protein encoded by the *rpmE2* gene might have an opposing role in ribosomal destabilization, that is, to induce a stronger translation abortion at *mgtQ* than that observed in wild type. The proposed function of the *rpmE2* gene seemed not to be exerted via Asp2 and Glu5 residues in *mgtQ* because the *rpmE2* mutation further increased the Asp2- and Glu5-substituted *mgtQ*′-*gfp* compared to the wild-type *mgtQ′-gfp* (Fig. 6C). The facilitated *mgtQ* translation of both the wild-type and 2,5Ala-substituted *mgtQ* in the *rpmE2* mutant further decreased *mgtB′-gfp* levels, supporting the finding that *mgtQ* and *mgtB* translation are inversely correlated. Finally, the effect of the *rpmE1* mutation on determining *mgtQ* translation efficiency was dominant over that of the *rpmE2* mutation, because the *rpmE1 rpmE2* double mutant behaved just like the *rpmE1* mutant (Fig. 6C).

The data described above suggest that availability of the L31 ribosomal protein could control MgtB Mg^2+^ transporter production via *mgtQ* ORF translation. To further confirm whether we could tune MgtB production by controlling the L31 ribosomal protein levels, we used the chromosomal *rpmE1* deletion mutant and provided a plasmid with the *rpmE1* gene expressed from an arabinose-inducible promoter (see Fig. S7). Similar to those detected in *mgtB′-gfp* levels, the MgtB protein levels were lower in the *rpmE1* deletion mutant than those in the wild type (see Fig. S7). However, as we increased the L31 protein levels by adding arabinose, MgtB protein levels started to be restored to those of wild type at 1 mM arabinose and increased further at 10 mM arabinose (see Fig. S7), indicating that MgtB Mg^2+^ transporter production is indeed controlled by the cellular availability of the L31 ribosomal proteins.

10.1128/mBio.03376-20.8 FIG S7 Tuning L31 protein production restores MgtB production. (A and B) Western blot analysis of crude extracts prepared from wild-type or the *rpmE1* mutant (EN1119) *Salmonella* harboring the *rpmE1* gene from an arabinose inducible promoter (pBAD-*rpmE1*) probed with anti-MgtB (A) or anti-Fur (B) antibodies to detect MgtB or Fur proteins, respectively. Bacteria were grown for 5 h in N-minimal media containing 0.01 mM Mg^2+^ in the presence of the indicated concentration of arabinose. Download FIG S7, TIF file, 0.09 MB. Copyright © 2021 Choi et al. 2021 Choi et al. https://creativecommons.org/licenses/by/4.0/ This content is distributed under the terms of the Creative Commons Attribution 4.0 International license.

### Acidic residues in *mgtQ* are required for promoting MgtB Mg^2+^ transporter production in low Mg^2+^.

AS described above, in the wild type, *mgtQ*-translating ribosomes are likely to be destabilized at Asp and Glu codons in *mgtQ* and allow the formation of stem-loop 2:3, thereby releasing the RBS of the *mgtB* gene to be translated. The degree of ribosome destabilization could be greater in ribosomes lacking the *rpmE1-*encoded L31 subunit, thus further promoting *mgtB* translation.

**FIG 7:**
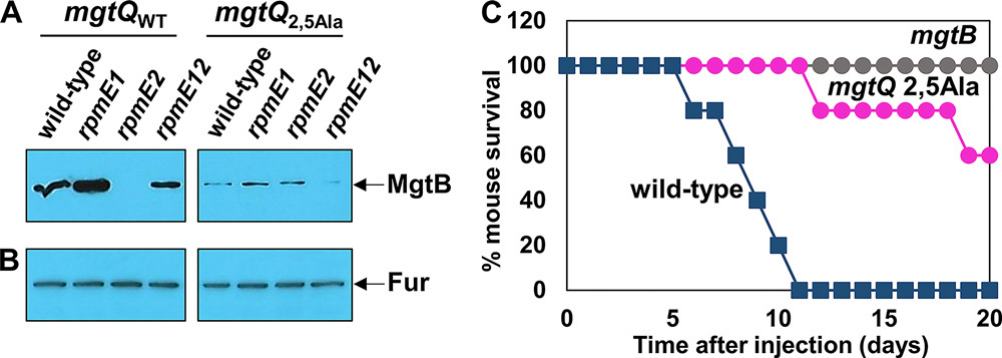
*mgtQ* translation promotes MgtB production and is required for *Salmonella* virulence in mice. (A and B) Western blot analysis of crude extracts prepared from wild-type, *rpmE1*, *rpmE2*, or *rpmE1 rpmE2 Salmonella* strains with either the wild-type *mgtQ* (14028s, EN1119, EN1120, or EN1368, respectively) or *mgtQ*_2,5Ala_ (EN1389, EN1408, EN1409, or EN1414, respectively) gene probed with anti-MgtB (A) or anti-Fur (B) antibodies to detect MgtB or Fur proteins, respectively. Bacteria were grown for 5 h in N-minimal media containing 0.01 mM Mg^2+^ as described in Materials and Methods. (C) The *mgtQ*_2,5Ala_ substitution attenuates *Salmonella* virulence in mice. Survival of C3H/HeN mice inoculated intraperitoneally with approximately 3000 CFU of wild-type (14028s), *mgtQ*_2,5Ala_ mutant (EN1389), and *mgtB* deletion mutant (EN481) *Salmonella* strains.

To understand the physiological role of the translation-aborting *mgtQ* ORF, we created a chromosomal *mgtQ* mutant with Asp2 and Glu5 substituted to Ala in the wild-type, *rpmE1*, *rpmE2*, or *rpmE1 rpmE2* deletion backgrounds. In wild-type *Salmonella*, MgtB proteins were detected when grown in low Mg^2+^ to activate transcription from the PhoP-dependent promoter (19) (Fig. 7A). However, the Asp2-to-Ala and Glu5-to-Ala substitutions in *mgtQ* severely decreased MgtB production (Fig. 7A). This could be ascribed to the efficient *mgtQ* translation mediated by the Asp2-to-Ala and Glu5-to-Ala substitutions (Fig. 6C; see also Fig. S5) because efficient *mgtQ* translation likely favors the formation of stem-loops 1:2 and 3:4 instead of stem-loop 2:3. *rpmE1* deletion seems to strengthen the ribosomal destabilization at *mgtQ* because it further increased MgtB protein levels (Fig. 7A). In contrast, *rpmE2* deletion completely abolished MgtB production (Fig. 7A) by promoting *mgtQ* translation (Fig. 6C). Combining *rpmE1* and *rpmE2* mutations restored MgtB protein levels similar to those in the wild type, suggesting that two types of L31 ribosomal proteins have opposing effects on ribosomal destabilization at *mgtQ* (Fig. 7A). The Asp2 and Glu5 codons at *mgtQ* are critical for determining *mgtQ* translation efficiency and thus control *mgtB* translation because when we substituted the Asp2 and Glu5 in *mgtQ* to Ala codons, MgtB protein levels were low, regardless of the *rpmE1* or *rpmE2* mutations (Fig. 7A). As a control experiment, Fur protein levels were unaffected in all tested conditions (Fig. 7B).

### The Asp2 and Glu5 codons at *mgtQ* are required for *Salmonella* virulence by ensuring MgtB production.

The *mgtB* gene is required for *Salmonella* virulence in Nramp1 (natural resistance-associated macrophage protein 1)-expressing mice (20). Given that the Asp2 and Glu5 to Ala substitution in *mgtQ* decreased MgtB protein levels (Fig. 7A), we tested whether the Asp2 and Glu5 codons in *mgtQ* would be required for *Salmonella* virulence by controlling MgtB protein production. When approximately 3,000 CFU of *Salmonella* were injected intraperitoneally in C3H/HeN Nramp1^+/+^ mice, the *Salmonella* strain with the Asp2 and Glu5 to Ala substitution in *mgtQ* was attenuated for virulence in mice compared to wild type (Fig. 7C). As a control, a strain lacking the *mgtB* gene was completely defective for virulence in mice (Fig. 7C). Therefore, these data indicate that the Asp2- and Glu5-mediated ribosomal destabilization at *mgtQ* guarantees MgtB Mg^2+^ transporter production, which might be critical for *Salmonella* pathogenesis by acquiring Mg^2+^ ions during infection.

## DISCUSSION

Here, we discovered *mgtQ*, an 8-amino-acid (aa) ORF in the *mgtCBRU* operon in S. enterica. The *mgtQ* ORF is located between the *mgtC* and *mgtB* genes and is required for downstream MgtB Mg^2+^ transporter production. The *mgtQ* ORF harbors Asp and Glu codons at positions 2 and 5, respectively, which intrinsically impede translation (see Fig. S5). The Asp- and Glu-mediated decrease in *mgtQ* translation reflects that *mgtQ*-translating ribosomes might be destabilized at *mgtQ*. In turn, this decrease in *mgtQ* translation efficiency promotes MgtB production possibly because it favors the formation of a stem-loop structure that suppresses the formation of a mutually exclusive translation-inhibitory stem-loop structure near the RBS of the *mgtB* gene. Thus, the substitution of Asp and Glu codons in *mgtQ* decreased MgtB Mg^2+^ transporter production and attenuated virulence in mice by affecting MgtB-mediated *Salmonella*’s ability to import Mg^2+^ ions during infection.

Conservation of *mgtQ* among several bacteria harboring the *mgtC* and *mgtB* genes in the same transcription unit (see Fig. S2) suggests that *mgtQ* might be required for a condition in which both the MgtC virulence protein and MgtB Mg^2+^ transporter act together. However, the genomic distribution of the *mgtQ* ORF in other bacteria also suggests that the *mgtQ* ORF is linked to the *mgtB* gene. In Brucella melitensis, the *mgtB* gene precedes the *mgtC* gene and is also separated by two other genes. In this case, we found an *mgtQ*-like ORF upstream of the *mgtB* gene (see Fig. S2). Mycobacterium tuberculosis contains the *mgtC* gene, but not the *mgtB* gene. As expected, we could not find an *mgtQ* ortholog in this strain (see Fig. S2). Together with our findings showing that *mgtQ* affects translation of the *mgtB* gene, the distribution suggests that *mgtQ* could be a *cis*-acting regulatory element that guarantees MgtB Mg^2+^ transporter production.

Translation initiation regions in mRNAs are often folded into a secondary structure that occludes ribosome binding and inhibits translation. To initiate protein synthesis, such a ribosome-inaccessible secondary structure needs to be destabilized to allow the ribosome to bind the Shine-Dalgarno sequence and/or the start codon. This could be achieved by a variety of mechanisms that release the RBS/start codon. RNA-binding proteins and/or small regulatory RNAs can bind the anti-RBS sequence to release the RBS site or an endonucleolytic cleavage could remove upstream anti-RBS sequence that was base-paired to the RBS and allow the ribosome to access the RBS (4, 6). Small ligands could bind to the mRNA and promote to form an alternative RNA secondary structure that releases the RBS from the translation-inhibitory stem-loop structure (6, 21). In this example, translation of the short ORF *mgtQ* also contributes to destabilizing a translation-inhibitory stem-loop structure of the downstream *mgtB* gene, similarly to a transcription attenuation mechanism found in the E. coli *trp* operon (22). However, the *mgtQ*-mediated translational control differs from that of *trpL* in the *trp* operon because the *mgtQ* ORF and its neighboring sequence are associated with a translation-inhibitory stem-loop structure, while the *trpL* leader peptide gene is associated with a Rho-independent transcriptional terminator (22). Moreover, *mgtQ* is located in the intergenic region of the first *mgtC* gene and the second *mgtB* gene within the *mgtCBRU* operon. In contrast, most known transcription attenuators, including the *trp* operon, are found in the 5′ leader regions of the operons (22, 23).

During *mgtQ* translation, the *mgtQ*-translating ribosome occupies an mRNA sequence overlapping *mgtQ* (region 1) and allows the formation of stem-loop structure 2:3, which sequesters the anti-RBS sequence and releases the RBS of the *mgtB* gene. The *mgtQ*-mediated *mgtB* RBS release is further enhanced by the codon composition of the *mgtQ* ORF because *mgtQ* contains Asp and Glu codons that are known to induce ribosome destabilization (18). Given that the ribosomal subunit L31 could counteract the Asp and Glu codon-mediated ribosome destabilization (18), *mgtQ*’s effect on *mgtB* translation could be maximized in a strain lacking the *rpmE1* gene (Fig. 6 and 7) or a condition that decreases the availability of the L31 subunit encoded by the *rpmE1* gene.

A translating ribosome can stall or be destabilized on mRNA depending on mRNA sequences. Two or more proline codons on mRNA induce ribosome stalling due to the intrinsic difficulty in forming a peptide bond when peptidyl-prolyl-tRNA at the P-site transfers a peptidyl-prolyl group to an incoming prolyl-tRNA at the A-site (24, 25). In this case, elongation factor EF-P rescues the stalled ribosome to continue translation (24, 25). When cellular free tryptophan levels are high, expression of the *tna* operon encoding tryptophanase in E. coli is induced by a mechanism that also involves yet another type of ribosome stalling (26). A ribosome translating the TnaC leader peptide in the *tna* operon stalls when the ribosome reaches the last Pro sense codon in the P-site and UGA stop codon in the A-site (27). In this case, Trp codon at position 12 of *tnaC* is required for the ribosome stalling because Trp12 in the nascent peptide binds to the ribosome exit tunnel and blocks the nascent peptide release at the *tnaC* UGA stop codon and ribosome dissociation. This stalling prevents Rho loading and thus Rho-dependent transcription termination, thus increasing *tna* operon expression. In addition, the stalling can be eventually relieved by release factor RF-3 and ribosome recycling factor RRF (26). Repeated acidic Asp and Glu codons on mRNA also induce ribosome destabilization and subsequent translation abortion because Asp and Glu residues on a nascent peptide interact with the interior of the exit tunnel (18). In this case, the ribosomal subunit L31 counteracts the destabilized ribosome to continue translation on mRNA. Interestingly, *Salmonella* harbors two L31 subunits encoded by the *rpmE1* and *rpmE2* genes, respectively. Based on our results that *rpmE1* and *rpmE2* mutations showed opposing effects on *mgtQ′-gfp* (Fig. 6) and reports from others that one of two L31 subunits is expressed in Zn^2+^-depleted media (28), the functional roles of two L31 subunits might differ.

Finally, the *mgtCBRU* operon contains several small ORFs including the third *mgtR* and fourth *mgtU* genes encoding 30- and 28-aa regulatory peptides that guide MgtC and MgtB to the FtsH-mediated proteolysis, respectively (29, 30). The 12-aa *mgtM* and 16-aa *mgtP* ORFs are located in the 5′ leader region of the operon and mediate ATP- and charged tRNA^Pro^-responsive transcriptional control of the *mgtCBRU* operon, respectively (31, 32). The 8-aa *mgtQ* is located in the *mgtC-mgtB* intergenic region and controls *mgtB* translation via a mechanism similar to acidic residue-mediated ribosomal destabilization. Although it is not clear why the *mgtCBRU* operon harbors multiple small ORFs to control expression of the entire operon or a part of the operon, *Salmonella* seems to have evolved to fine-tune the levels of proteins within this virulence operon in response to different signals, including those from the host environment.

## MATERIALS AND METHODS

### Bacterial strains, plasmids, oligodeoxynucleotides, and growth conditions.

Bacterial strains and plasmids used in this study are listed in Table S1 in the supplemental material. All S. enterica serovar Typhimurium strains were derived from the wild-type strain 14028s (33) and were constructed by one-step gene inactivation method (34) and/or P22-mediated transduction as previously described (35). DNA oligonucleotides are listed in Table S2. Bacteria were grown at 37°C in Luria-Bertani broth, N-minimal media (36) supplemented with 0.1% Casamino Acids and 38 mM glycerol, and the indicated concentrations of MgCl_2_. E. coli DH5α was used as the host for preparing plasmid DNA. Ampicillin was used at 50 μg ml^−1^, chloramphenicol was used at 20 μg ml^−1^, kanamycin was used at 20 μg ml^−1^, tetracycline was used at 10 μg ml^−1^, fusaric acid (37) was used at 12 μg ml^−1^, and l-arabinose was used at 0.2% (wt/vol). See Text S1 for more information.

10.1128/mBio.03376-20.9 TABLE S1 Bacterial strains and plasmids used in this study. Download Table S1, DOCX file, 0.04 MB. Copyright © 2021 Choi et al. 2021 Choi et al. https://creativecommons.org/licenses/by/4.0/ This content is distributed under the terms of the Creative Commons Attribution 4.0 International license.

10.1128/mBio.03376-20.10 TABLE S2 Primers used in this study. Download Table S2, DOCX file, 0.03 MB. Copyright © 2021 Choi et al. 2021 Choi et al. https://creativecommons.org/licenses/by/4.0/ This content is distributed under the terms of the Creative Commons Attribution 4.0 International license.

### Data availability.

All other relevant data are available from the corresponding author upon reasonable request.

10.1128/mBio.03376-20.1 TEXT S1 Supplemental materials and methods. Download Text S1, DOCX file, 0.04 MB. Copyright © 2021 Choi et al. 2021 Choi et al. https://creativecommons.org/licenses/by/4.0/ This content is distributed under the terms of the Creative Commons Attribution 4.0 International license.

## References

[1] Govantes F, Andujar E, Santero E. 1998. Mechanism of translational coupling in the nifLA operon of *Klebsiella pneumoniae*. EMBO J 17:2368–2377. doi:10.1093/emboj/17.8.2368. 9545248 PMC1170580

[2] Koonin EV, Wolf YI. 2008. Genomics of bacteria and archaea: the emerging dynamic view of the prokaryotic world. Nucleic Acids Res 36:6688–6719. doi:10.1093/nar/gkn668. 18948295 PMC2588523

[3] Thorpe HA, Bayliss SC, Hurst LD, Feil EJ. 2017. Comparative analyses of selection operating on nontranslated intergenic regions of diverse bacterial species. Genetics 206:363–376. doi:10.1534/genetics.116.195784. 28280056 PMC5419481

[4] McCarthy JE, Gualerzi C. 1990. Translational control of prokaryotic gene expression. Trends Genet 6:78–85. doi:10.1016/0168-9525(90)90098-q. 2183416

[5] Desnoyers G, Bouchard MP, Masse E. 2013. New insights into small RNA-dependent translational regulation in prokaryotes. Trends Genet 29:92–98. doi:10.1016/j.tig.2012.10.004. 23141721

[6] Geissmann T, Marzi S, Romby P. 2009. The role of mRNA structure in translational control in bacteria. RNA Biol 6:153–160. doi:10.4161/rna.6.2.8047. 19885993

[7] Oppenheim DS, Yanofsky C. 1980. Translational coupling during expression of the tryptophan operon of *Escherichia coli*. Genetics 95:785–795. 6162715 10.1093/genetics/95.4.785 PMC1214269

[8] Lee EJ, Groisman EA. 2010. An antisense RNA that governs the expression kinetics of a multifunctional virulence gene. Mol Microbiol 76:1020–1033. doi:10.1111/j.1365-2958.2010.07161.x. 20398218 PMC2909850

[9] Yeom J, Pontes MH, Choi J, Groisman EA. 2018. A protein that controls the onset of a *Salmonella* virulence program. EMBO J 37. doi:10.15252/embj.201796977. PMC6043847 29858228

[10] Smith DL, Tao T, Maguire ME. 1993. Membrane topology of a P-type ATPase: the MgtB magnesium transport protein of *Salmonella* Typhimurium. J Biol Chem 268:22469–22479. doi:10.1016/S0021-9258(18)41553-0. 8226755

[11] Soncini FC, Garcia Vescovi E, Solomon F, Groisman EA. 1996. Molecular basis of the magnesium deprivation response in *Salmonella* Typhimurium: identification of PhoP-regulated genes. J Bacteriol 178:5092–5099. doi:10.1128/jb.178.17.5092-5099.1996. 8752324 PMC178303

[12] Lee EJ, Pontes MH, Groisman EA. 2013. A bacterial virulence protein promotes pathogenicity by inhibiting the bacterium’s own F1Fo ATP synthase. Cell 154:146–156. doi:10.1016/j.cell.2013.06.004. 23827679 PMC3736803

[13] Maguire ME, Cowan JA. 2002. Magnesium chemistry and biochemistry. Biometals 15:203–210. doi:10.1023/a:1016058229972. 12206387

[14] Wacker WE. 1969. The biochemistry of magnesium. Ann N Y Acad Sci 162:717–726. doi:10.1111/j.1749-6632.1969.tb13003.x. 4242156

[15] Molina N, van Nimwegen E. 2008. Universal patterns of purifying selection at noncoding positions in bacteria. Genome Res 18:148–160. doi:10.1101/gr.6759507. 18032729 PMC2134783

[16] Tsai CH, Liao R, Chou B, Palumbo M, Contreras LM. 2015. Genome-wide analyses in bacteria show small-RNA enrichment for long and conserved intergenic regions. J Bacteriol 197:40–50. doi:10.1128/JB.02359-14. 25313390 PMC4288687

[17] Zuker M. 2003. Mfold web server for nucleic acid folding and hybridization prediction. Nucleic Acids Res 31:3406–3415. doi:10.1093/nar/gkg595. 12824337 PMC169194

[18] Chadani Y, Niwa T, Izumi T, Sugata N, Nagao A, Suzuki T, Chiba S, Ito K, Taguchi H. 2017. Intrinsic ribosome destabilization underlies translation and provides an organism with a strategy of environmental sensing. Mol Cell 68:528–539 e5. doi:10.1016/j.molcel.2017.10.020. 29100053

[19] Zwir I, Shin D, Kato A, Nishino K, Latifi T, Solomon F, Hare JM, Huang H, Groisman EA. 2005. Dissecting the PhoP regulatory network of *Escherichia coli* and *Salmonella enterica*. Proc Natl Acad Sci U S A 102:2862–2867. doi:10.1073/pnas.0408238102. 15703297 PMC548500

[20] Choi E, Choi S, Nam D, Park S, Han Y, Lee JS, Lee EJ. 2017. Elongation factor P restricts *Salmonella*’s growth by controlling translation of a Mg^2+^ transporter gene during infection. Sci Rep 7:42098. doi:10.1038/srep42098. 28181542 PMC5299641

[21] Breaker RR. 2018. Riboswitches and translation control. Cold Spring Harb Perspect Biol 10:a032797. doi:10.1101/cshperspect.a032797. 29844057 PMC6211393

[22] Merino E, Yanofsky C. 2005. Transcription attenuation: a highly conserved regulatory strategy used by bacteria. Trends Genet 21:260–264. doi:10.1016/j.tig.2005.03.002. 15851059

[23] Naville M, Gautheret D. 2010. Transcription attenuation in bacteria: theme and variations. Brief Funct Genomics 9:178–189. doi:10.1093/bfgp/elq008. 20352660

[24] Ude S, Lassak J, Starosta AL, Kraxenberger T, Wilson DN, Jung K. 2013. Translation elongation factor EF-P alleviates ribosome stalling at polyproline stretches. Science 339:82–85. doi:10.1126/science.1228985. 23239623

[25] Doerfel LK, Wohlgemuth I, Kothe C, Peske F, Urlaub H, Rodnina MV. 2013. EF-P is essential for rapid synthesis of proteins containing consecutive proline residues. Science 339:85–88. doi:10.1126/science.1229017. 23239624

[26] Gong F, Ito K, Nakamura Y, Yanofsky C. 2001. The mechanism of tryptophan induction of tryptophanase operon expression: tryptophan inhibits release factor-mediated cleavage of TnaC-peptidyl-tRNA^Pro^. Proc Natl Acad Sci U S A 98:8997–9001. doi:10.1073/pnas.171299298. 11470925 PMC55362

[27] Gong F, Yanofsky C. 2002. Instruction of translating ribosome by nascent peptide. Science 297:1864–1867. doi:10.1126/science.1073997. 12228716

[28] Nanamiya H, Akanuma G, Natori Y, Murayama R, Kosono S, Kudo T, Kobayashi K, Ogasawara N, Park SM, Ochi K, Kawamura F. 2004. Zinc is a key factor in controlling alternation of two types of L31 protein in the *Bacillus subtilis* ribosome. Mol Microbiol 52:273–283. doi:10.1111/j.1365-2958.2003.03972.x. 15049826

[29] Alix E, Blanc-Potard AB. 2008. Peptide-assisted degradation of the *Salmonella* MgtC virulence factor. EMBO J 27:546–557. doi:10.1038/sj.emboj.7601983. 18200043 PMC2241655

[30] Yeom J, Shao Y, Groisman EA. 2020. Small proteins regulate *Salmonella* survival inside macrophages by controlling degradation of a magnesium transporter. Proc Natl Acad Sci U S A 117:20235–20243. doi:10.1073/pnas.2006116117. 32753384 PMC7443967

[31] Lee EJ, Groisman EA. 2012. Control of a *Salmonella* virulence locus by an ATP-sensing leader messenger RNA. Nature 486:271–275. doi:10.1038/nature11090. 22699622 PMC3711680

[32] Lee EJ, Groisman EA. 2012. Tandem attenuators control expression of the *Salmonella mgtCBR* virulence operon. Mol Microbiol 86:212–224. doi:10.1111/j.1365-2958.2012.08188.x. 22857388 PMC3641672

[33] Fields PI, Swanson RV, Haidaris CG, Heffron F. 1986. Mutants of *Salmonella* Typhimurium that cannot survive within the macrophage are avirulent. Proc Natl Acad Sci U S A 83:5189–5193. doi:10.1073/pnas.83.14.5189. 3523484 PMC323916

[34] Datsenko KA, Wanner BL. 2000. One-step inactivation of chromosomal genes in *Escherichia coli* K-12 using PCR products. Proc Natl Acad Sci U S A 97:6640–6645. doi:10.1073/pnas.120163297. 10829079 PMC18686

[35] Davis RW, Bolstein D, Roth JR. 1980. Advanced bacterial genetics. Cold Spring Harbor Laboratories, Cold Spring Harbor, NY.

[36] Snavely MD, Miller CG, Maguire ME. 1991. The *mgtB* Mg^2+^ transport locus of *Salmonella* Typhimurium encodes a P-type ATPase. J Biol Chem 266:815–823. doi:10.1016/S0021-9258(17)35246-8. 1824701

[37] Maloy SR, Nunn WD. 1981. Selection for loss of tetracycline resistance by *Escherichia coli*. J Bacteriol 145:1110–1111. doi:10.1128/JB.145.2.1110-1111.1981. 7007341 PMC217228

