# Supplementary figures and images for: A Translation-Aborting Small Open Reading Frame in the Intergenic Region Promotes Translation of a Mg2+ Transporter in Salmonella Typhimurium
Source: mBio. 2021 Apr 13;12(2):e03376-20. doi: 10.1128/mBio.03376-20 (PMC8092293; doi:10.1128/mBio.03376-20)

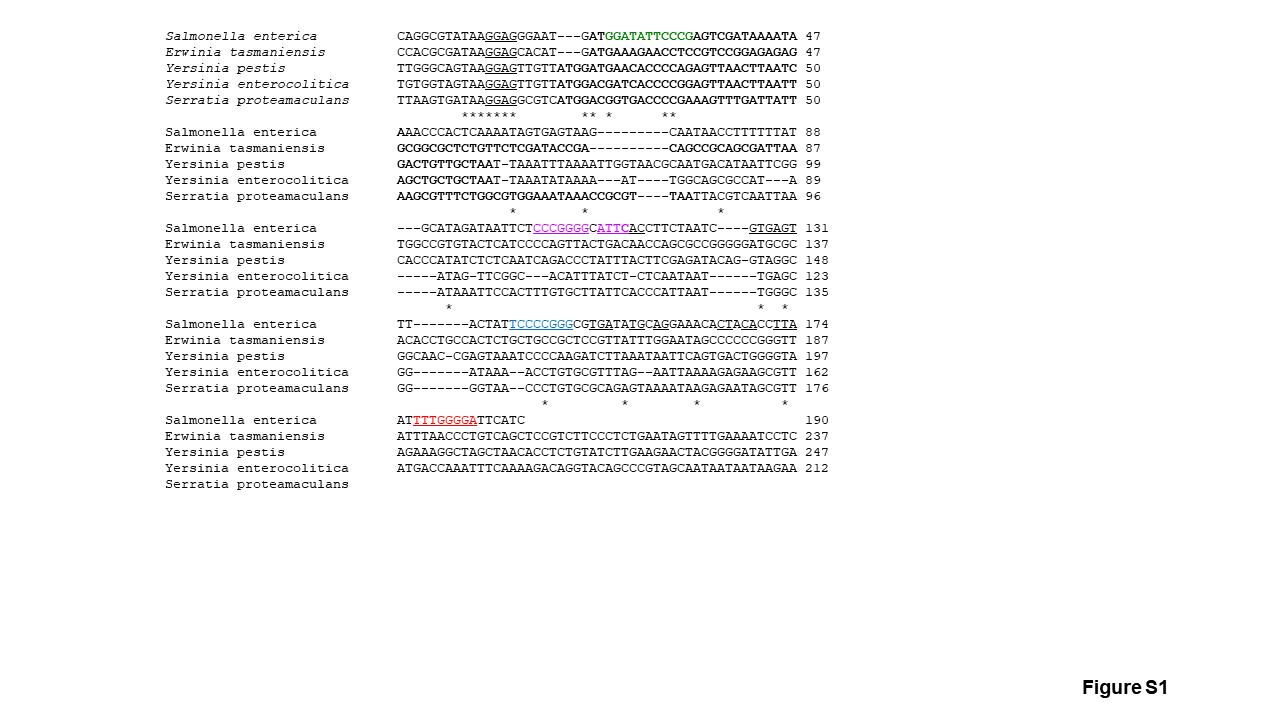

Supplement: FIG S1 [file mBio.03376-20-sf001.tif]

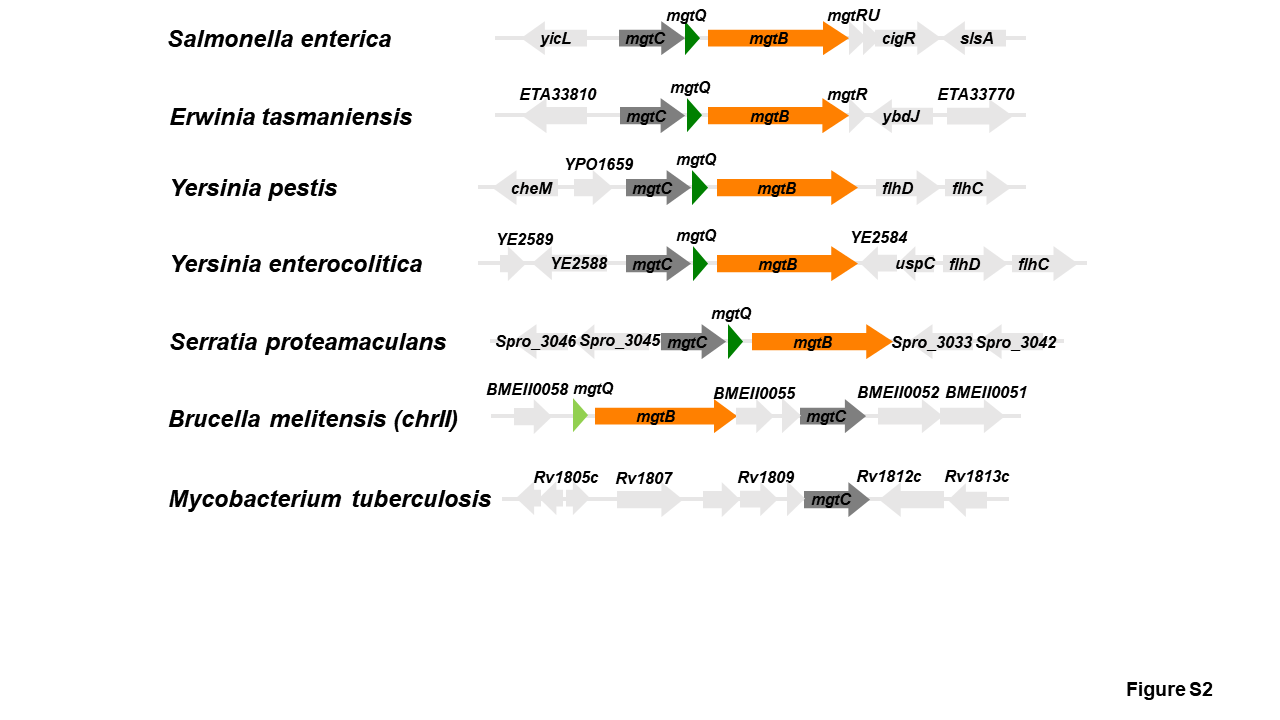

Supplement: FIG S2 [file mBio.03376-20-sf002.tif]

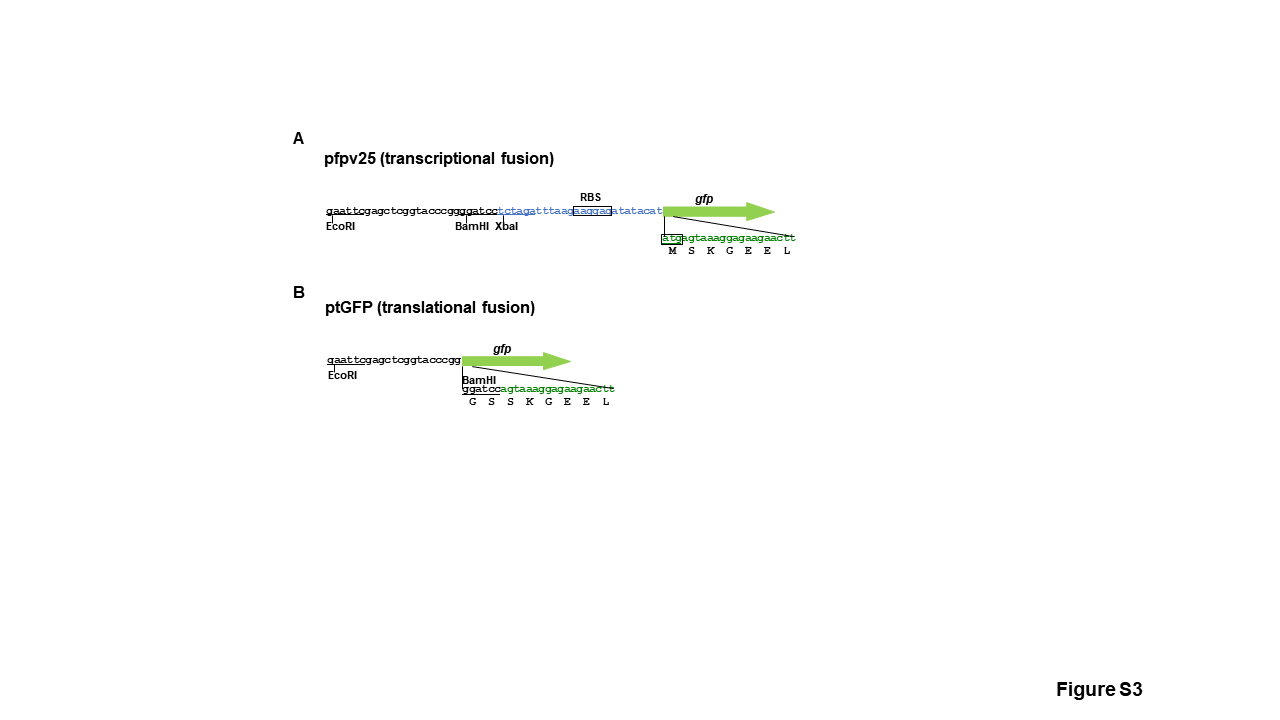

Supplement: FIG S3 [file mBio.03376-20-sf003.tif]

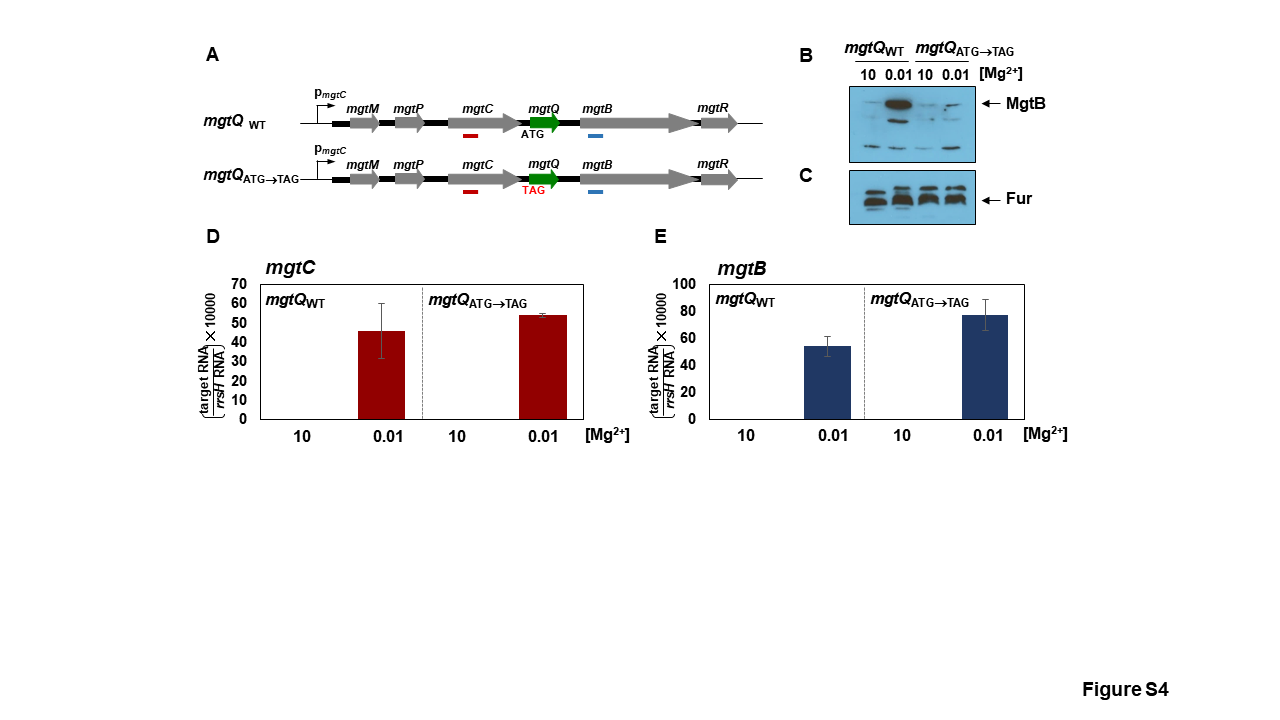

Supplement: FIG S4 [file mBio.03376-20-sf004.tif]

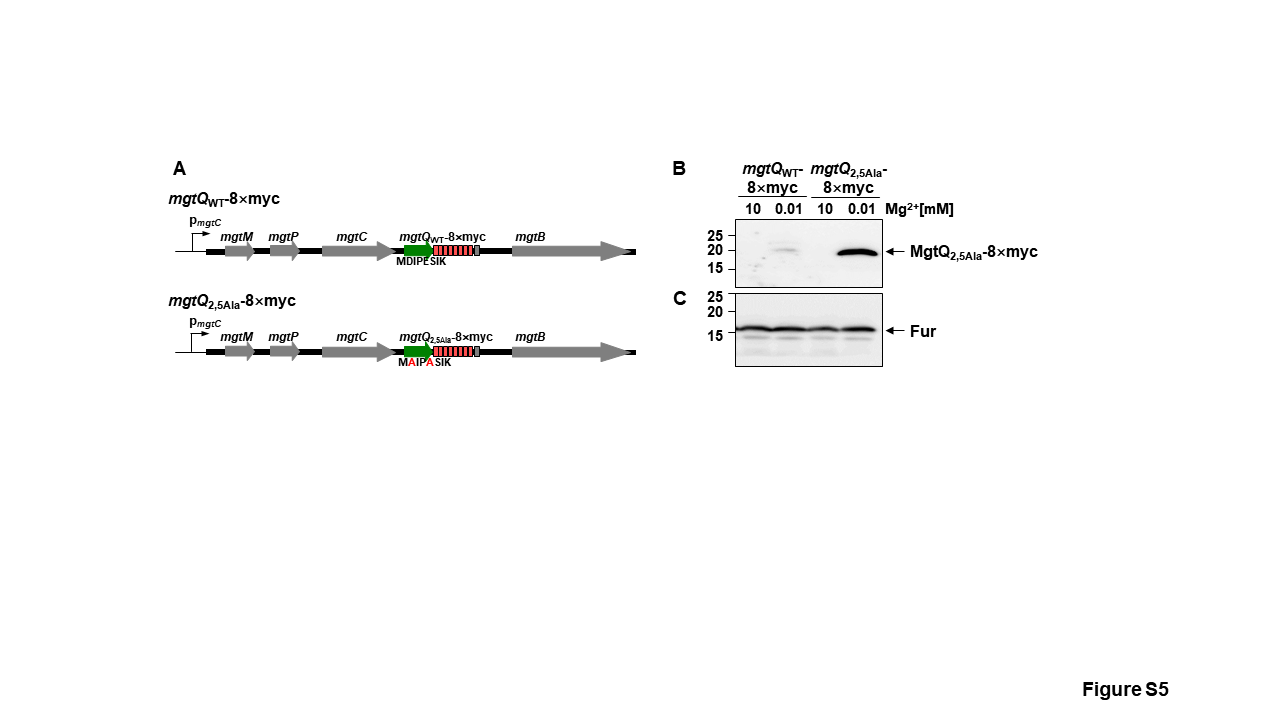

Supplement: FIG S5 [file mBio.03376-20-sf005.tif]

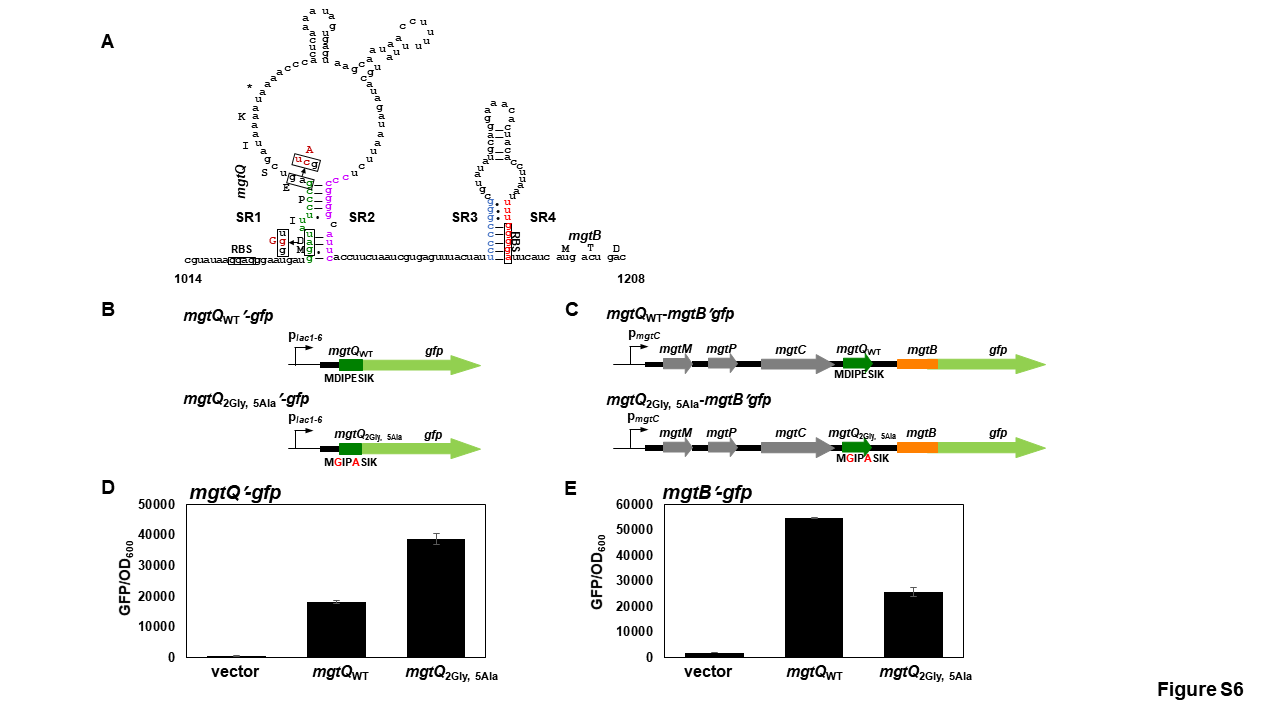

Supplement: FIG S6 [file mBio.03376-20-sf006.tif]

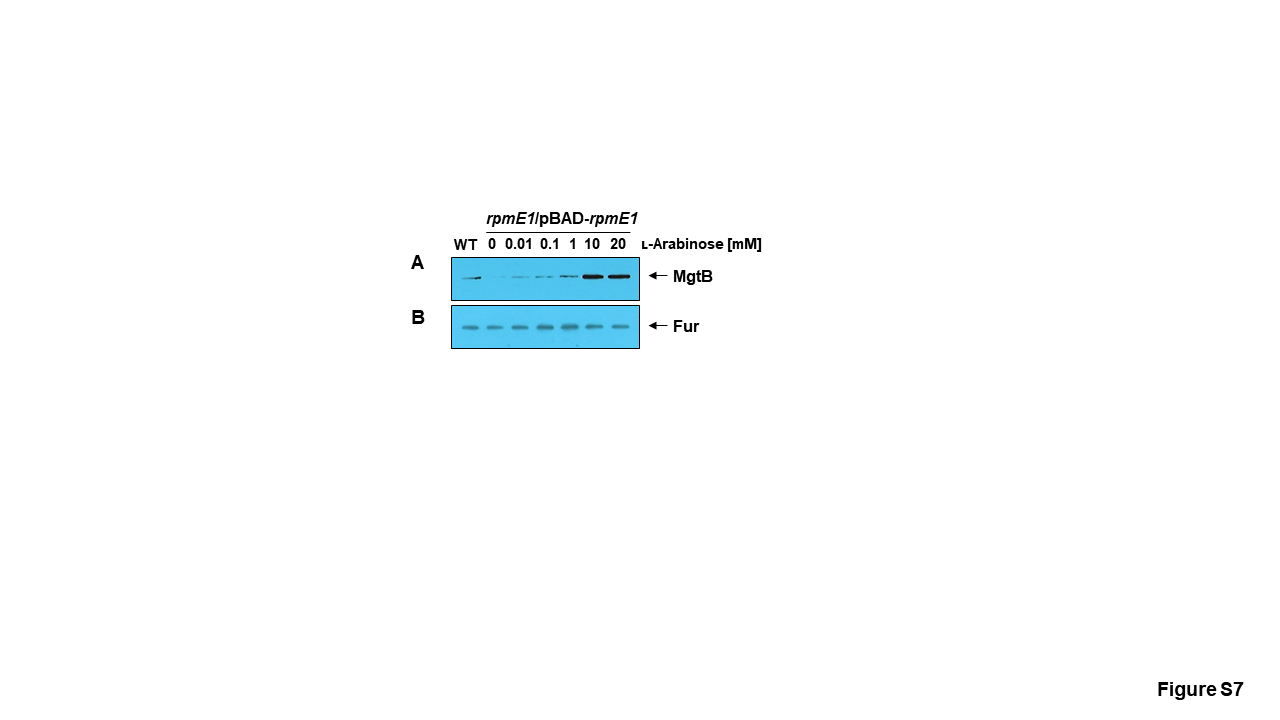

Supplement: FIG S7 [file mBio.03376-20-sf007.tif]
