# Supplementary material for: A Translation-Aborting Small Open Reading Frame in the Intergenic Region Promotes Translation of a Mg2+ Transporter in Salmonella Typhimurium
Source: mBio. 2021 Apr 13;12(2):e03376-20. doi: 10.1128/mBio.03376-20 (PMC8092293; doi:10.1128/mBio.03376-20)
Supplement: TABLE S1 [file mBio.03376-20-st001.docx]

**Table S1. Bacterial strains and plasmids used in this study**

| Strain or Plasmid | Description | Reference or Source |
| --- | --- | --- |
| ***S. enterica* serovar Typhimurium strains** | | |
| 14028s | wild-type | ([1](#_ENREF_1)) |
| EG18750 | [*mgtC-mgtB*]IG::Tet^R^ | ([2](#_ENREF_2)) |
| EG17087 | Cm^R^::p*_lac_*_1-6_-*mgtCBR* | This work |
| EG17098 | p*_lac_*_1-6_- *mgtCBR* | This work |
| EL626 | *mgtQ* (ATG→TAG) | This work |
| EN481 | *mgtB* | ([3](#_ENREF_3)) |
| DN584 | *rpmE1*::Km^R^ | This work |
| DN585 | *rpmE2*::Km^R^ | This work |
| EN1119 | *rpmE1* | This work |
| EN1120 | *rpmE2* | This work |
| DN337 | *efp*::Cm^R^ | ([4](#_ENREF_4)) |
| DN369 | *efp* | ([4](#_ENREF_4)) |
| EN1368 | *rpmE1, rpmE2* | This work |
| EN1389 | *mgtQ* (Asp2, Glu5→Ala) | This work |
| EN1408 | *mgtQ* (Asp2, Glu5→Ala), *rpmE1* | This work |
| EN1409 | *mgtQ* (Asp2, Glu5→Ala)*, rpmE2* | This work |
| EN1414 | *mgtQ* (Asp2, Glu5→Ala), *rpmE1 rpmE2* | This work |
| EN1465 | *mgtQ*-8×*myc*::Km^R^ | This work |
| EN1467 | *mgtQ* (Asp2, Glu5→Ala)-8×*myc*::Km^R^ | This work |
| EN1469 | *mgtQ*-8×*myc* | This work |
| EN1471 | *mgtQ* (Asp2, Glu5→Ala)-8×*myc* | This work |
| **Plasmids** |  |  |
| pCP20 | rep_pSC101_^ts^ Ap^R^ Cm^R^ *FLP*^+^ λ *c*I857^+^ | ([5](#_ENREF_5)) |
| pKD3 | repR_R6K_ Ap^R^ FRT Cm^R^ FRT | ([5](#_ENREF_5)) |
| pKD4 | repR_R6K_ Ap^R^ FRT Km^R^ FRT | ([5](#_ENREF_5)) |
| pKD46 | rep_pSC101_^ts^ Ap^R^ p*_araBAD_* γ β exo | ([5](#_ENREF_5)) |
| pFPV25 | rep_pMB1_ Ap^R^, promoterless *gfp* | ([6](#_ENREF_6)) |
| p*_lac_*_1-6_-GFP | pFPV25 p*_lac_*_1-6_-*gfp* | This work |
| pGFP-*mgtB* | pFPV25 p*_mgtC_*-*mgtC-mgtQ*(wild-type*)-mgtB*-′*gfp* | This work |
| pGFP-*mgtQ*_ATG→TAG_-*mgtB* | pFPV25 p*_mgtC_*-*mgtC-mgtQ*(ATG→TAG*)-mgtB*-′*gfp* | This work |
| pGFP-*mgtB* Stem region 1 | pGFP-*mgtB* [*mgtC-mgtB*]IG (CC_1041,1042_→GG) | This work |
| pGFP-*mgtB* Stem region 2 | pGFP-*mgtB* [*mgtC-mgtB*]IG (GG_1115,1116_→CC) | This work |
| pGFP-*mgtB* Stem region 3 | pGFP-*mgtB* [*mgtC-mgtB*]IG (CC_1151,1152_→GG) | This work |
| pGFP-*mgtB* Stem region 4 | pGFP-*mgtB* [*mgtC-mgtB*]IG (GG_1189,1190_→CC) | This work |
| pGFP-*mgtB* Stem 1:2 | pGFP-*mgtB* [*mgtC-mgtB*]IG (CC_1041,1042_→GG, GG_1115,1116_→CC) | This work |
| pGFP-*mgtB* Stem 2:3 | pGFP-*mgtB* [*mgtC-mgtB*]IG (GG_1115,1116_→CC, CC_1151,1152_→GG) | This work |
| pGFP-*mgtB* Stem 3:4 | pGFP-*mgtB* [*mgtC-mgtB*]IG (CC_1151,1152_→GG, GG_1189,1190_→CC) | This work |
| pGFP-*mgtB* *mgtQ*(CCC→CCG) | pGFP-*mgtB* *mgtQ* (CCC_Pro_→CCG_Pro_) | This work |
| pGFP-*mgtB* *mgtQ*(CCC→GGG) | pGFP-*mgtB* *mgtQ* (CCC_Pro_→GGG_Gly_) | This work |
| pGFP-*mgtB* *mgtQ*(CCC→CTC) | pGFP-*mgtB* *mgtQ* (CCC_Pro_→CTC_Leu_) | This work |
| pGFP-*mgtB* *mgtQ*(CCC→CAC) | pGFP-*mgtB* *mgtQ* (CCC_Pro_→CAC_His_) | This work |
| pGFP-*mgtB* *mgtQ*(CCC→CGC) | pGFP-*mgtB* *mgtQ* (CCC_Pro_→CGC_Arg_) | This work |
| ptGFP | ColE1 ori Ap^R^ ′*gfp* | ([7](#_ENREF_7)) |
| p*_lac_*_1-6_-′GFP | pFPV25 p*_lac_*_1-6_-′*gfp* | This work |
| ptGFP-*mgtB* | pFPV25 p*_mgtC_*-*mgtC-mgtQ*(wild-type*)-mgtB*′-*gfp* | This work |
| ptGFP-*mgtQ*_ATG→TAG_-*mgtB* | pFPV25 p*_mgtC_*-*mgtC-mgtQ*(ATG→TAG*)-mgtB*′-*gfp* | This work |
| ptGFP-*mgtB* Stem region 1 | ptGFP-*mgtB* [*mgtC-mgtB*]IG (CC_1041,1042_→GG) | This work |
| ptGFP-*mgtB* Stem region 2 | ptGFP-*mgtB* [*mgtC-mgtB*]IG (GG_1115,1116_→CC) | This work |
| ptGFP-*mgtB* Stem region 3 | ptGFP-*mgtB* [*mgtC-mgtB*]IG (CC_1151,1152_→GG) | This work |
| ptGFP-*mgtB* Stem region 4 | ptGFP-*mgtB* [*mgtC-mgtB*]IG (GG_1189,1190_→CC) | This work |
| ptGFP-*mgtB* Stem 1:2 | ptGFP-*mgtB* [*mgtC-mgtB*]IG (CC_1041,1042_→GG, GG_1115,1116_→CC) | This work |
| ptGFP-*mgtB* Stem 2:3 | ptGFP-*mgtB* [*mgtC-mgtB*]IG (GG_1115,1116_→CC, CC_1151,1152_→GG) | This work |
| ptGFP-*mgtB* Stem 3:4 | ptGFP-*mgtB* [*mgtC-mgtB*]IG (CC_1151,1152_→GG, GG_1189,1190_→CC) | This work |
| ptGFP-*mgtB* *mgtQ*(CCC→CCG) | ptGFP-*mgtB* *mgtQ* (CCC_Pro_→CCG_Pro_) | This work |
| ptGFP-*mgtB* *mgtQ*(CCC→GGG) | ptGFP-*mgtB* *mgtQ* (CCC_Pro_→GGG_Gly_) | This work |
| ptGFP-*mgtB* *mgtQ*(CCC→CTC) | ptGFP-*mgtB* *mgtQ* (CCC_Pro_→CTC_Leu_) | This work |
| ptGFP-*mgtB* *mgtQ*(CCC→CAC) | ptGFP-*mgtB* *mgtQ* (CCC_Pro_→CAC_His_) | This work |
| ptGFP-*mgtB* *mgtQ*(CCC→CGC) | ptGFP-*mgtB* *mgtQ* (CCC_Pro_→CGC_Arg_) | This work |
| ptGFP-*mgtB* (*mgtQ* (Asp2, Glu5→Ala) | ptGFP-*mgtB* *mgtQ* (GAT_Asp_, GAG_Glu_→GCT_Ala_) | This work |
| ptGFP-*mgtB* (*mgtQ* (Asp2→Gly, Glu5→Ala) | ptGFP-*mgtB* *mgtQ* (GAT_Asp_→GGT_Gly_, GAG_Glu_→GCT_Ala_) | This work |
| p*_lac1-6_*-*mgtQ*‘-GFP | pFPV25 p*_lac1-6_*-*mgtQ*‘-*gfp* | This work |
| p*_lac1-6_*-*mgtQ* _STOP_‘-GFP | pFPV25 p*_lac1-6_*-*mgtQ* stop codon‘-*gfp* | This work |
| p*_lac1-6_*-*mgtQ* _2,5 Ala_‘-GFP | pFPV25 p*_lac1-6_*- *mgtQ* (Asp2, Glu5→Ala)‘- *gfp* | This work |
| p*_lac1-6_*-*mgtQ* _2 Gly, 5 Ala_‘-GFP | pFPV25 p*_lac1-6_*- *mgtQ* (Asp2→Gly, Glu5→Ala)‘- *gfp* | This work |
| pBOP508 | repR_R6K_ Ap^R^ 8×myc FRT Km^R^ FRT | ([8](#_ENREF_8)) |
| pBAD33 | repR_p15_ Cm^R^ araC p_BAD_ promoter | ([9](#_ENREF_9)) |
| pBAD33-*rpmE1* | pBAD33 p_BAD_-*rpmE1* | This work |

##### References

1. Fields PI, Groisman EA, Heffron F. 1989. A *Salmonella* locus that controls resistance to microbicidal proteins from phagocytic cells. Science 243:1059-62.

2. Lee EJ, Groisman EA. 2010. An antisense RNA that governs the expression kinetics of a multifunctional virulence gene. Mol Microbiol 76:1020-33.

3. Choi E, Choi S, Nam D, Park S, Han Y, Lee JS, Lee EJ. 2017. Elongation factor P restricts Salmonella's growth by controlling translation of a Mg2+ transporter gene during infection. Sci Rep 7:42098.

4. Nam D, Choi E, Shin D, Lee EJ. 2016. tRNAPro-mediated downregulation of elongation factor P is required for *mgtCBR* expression during *Salmonella* infection. Mol Microbiol 102:221-232.

5. Datsenko KA, Wanner BL. 2000. One-step inactivation of chromosomal genes in *Escherichia coli* K-12 using PCR products. Proc Natl Acad Sci U S A 97:6640-5.

6. Valdivia RH, Falkow S. 1996. Bacterial genetics by flow cytometry: rapid isolation of *Salmonella typhimurium* acid-inducible promoters by differential fluorescence induction. Mol Microbiol 22:367-78.

7. Lee EJ, Groisman EA. 2012. Tandem Attenuators Control Expression of the *Salmonella* *mgtCBR* Virulence Operon. Mol Microbiol 86:212-24.

8. Cho BK, Knight EM, Palsson BO. 2006. PCR-based tandem epitope tagging system for Escherichia coli genome engineering. Biotechniques 40:67-72.

9. Guzman LM, Belin D, Carson MJ, Beckwith J. 1995. Tight regulation, modulation, and high-level expression by vectors containing the arabinose PBAD promoter. J Bacteriol 177:4121-30.
