# Supplementary material for: A Translation-Aborting Small Open Reading Frame in the Intergenic Region Promotes Translation of a Mg2+ Transporter in Salmonella Typhimurium
Source: mBio. 2021 Apr 13;12(2):e03376-20. doi: 10.1128/mBio.03376-20 (PMC8092293; doi:10.1128/mBio.03376-20)
Supplement: TABLE S2 [file mBio.03376-20-st002.docx]

##### Table S2. Primers used in this study

| **No.** | **Sequence (from 5′ to 3′)** |
| --- | --- |
| 1259 | GGGTTATTGAATGCCTGC |
| 7554 | CAATATCCGCTGAGGGAGAA |
| 6970 | CCAGCAGCCGCGGTAAT |
| 6971 | TTTACGCCCAGTAATTCCGATT |
| 7530 | CAGCCCGCGCACATTC |
| 7531 | TTGTCTCTGGGATTGGCTTTCT |
| 7763 | TCAGAAAATGATAAGCAGCATAAAAAA |
| 7764 | CCCTGACGATGGCTGTTCA |
| 7030 | ATTGTTTAATGATTTCAGACGAGCTTGTTATCGACATAATTGTAGGCTGGAGCTGCTTCG |
| 7031 | TAAAAGTGACTGTCCAACATATTACTCCTGCATGAATTTGTCCACACAACATAAACATAAAAAGCTTAAAGTGTAAAGCATATGAATATCCTCCTTAG |
| 9903 | CGGAATTCCTTTACACTTTAAGCTTTTTATG |
| 9904 | GCTCTAGAGGATCCCCGGGTACCGAGCTCATTACTCCTGCATGAATTTG |
| 10798 | AGGCGTATAAGGAGGGATAGTAGGATATTCCCGAGTCGAT |
| 10799 | ATCGACTCGGGAATATCCTACTATCCCTCCTTATACGCCT |
| 1746 | GGAATTCCTTTGCTCCATGATGTAC |
| mgtB27BR | CGCGGATCCCCGGTTTTCAATGTTCATGTC |
| KH019 | AATTCCTTTACACTTTAAGCTTTTTATGTTTATGTTGTGTGGAAGAAAAAATGGAGGAACGTATGTTAG |
| KH020 | GATCCTAACATACGTTCCTCCATTTTTTCTTCCACACAACATAAACATAAAAAGCTTAAAGTGTAAAGG |
| KH385 | ATG GAT ATT GGG GAG TCG ATA |
| KH389 | TAT CGA CTC CCC AAT ATC CAT |
| KH382 | AAT TCT CCC CCG GCA TTC AC |
| KH386 | GTG AAT GCC GGG GGA GAA TT |
| KH383 | TAC TAT TCC GGG GGC GTG AT |
| KH387 | ATC ACG CCC CCG GAA TAG TA |
| KH384 | CTT AAT TTT CCG GAT TCA TC |
| KH388 | GAT GAA TCC GGA AAA TTA AG |
| KH390 | AATTCTCCCCCGGCATTCACCTTCTAATCGTGAGTTTACTATTCCGGGGGCGTGAT |
| KH392 | ATCACGCCCCCGGAATAGTAAACTCACGATTAGAAGGTGAATGCCGGGGGAGAATT |
| KH391 | TAC TAT TCC GGG GGC GTG ATA TGC AGG AAA CAC TAC ACC TTA ATT TTC CGG ATT CAT C |
| KH393 | GAT GAA TCC GGA AAA TTA AGG TGT AGT GTT TCC TGC ATA TCA CGC CCC CGG AAT AGT A |
| KH429 | ATG GAT ATT CCG GAG TCG ATA |
| KH430 | TAT CGA CTC CGG AAT ATC CAT |
| KH922 | ATG GAT ATT CTC GAG TCG ATA |
| KH923 | TAT CGA CTC GAG AAT ATC CAT |
| KH431 | ATG GAT ATT CAC GAG TCG ATA |
| KH432 | TAT CGA CTC GTG AAT ATC CAT |
| KH433 | ATG GAT ATT CGC GAG TCG ATA |
| KH434 | TAT CGA CTC GCG AAT ATC CAT |
| stem A mut-F | ATG GAT ATT CGG GAG TCG ATA |
| stem A mut-R | TAT CGA CTC CCG AAT ATC CAT |
| KU34-mgtQ(2,5 ala)-F | GGA ATG ATG GCT ATT CCC GCT TCG ATA AAA |
| KU35-mgtQ(2,5 Ala)-R | TTT TAT CGA AGC GGG AAT AGC CAT CAT TCC |
| KH073 | GGAATTCCTTTACACTTTAAGCTTTTTATGTTTATGTTGTGTGGAAGTTTTTTATTCAATTGCAA |
| KH074 | CGGGATCCTTTTATCGACTCGGGAATAT |
| KH075 | CGGGATCCTTATTTTATCGACTCGGGAA |
| KU37 Rev-mgtQ(2,5 ala)-BamHI-new | CGGGATCCTTTTATCGAAGCGGGAATAGCCATCATTCC |
| KU53 Rev-mgtQ(ATG)-BamHI | CGCGGATCCTTTTATCGACTCGGGAATATCCTACTATCCCTCCTT |
| Del-rpmE-F | AGG GCT GGA AGA GCG ACG CGG CCT TAC ACT GAG GTT CCC CTG TAG GCT GGA GCT GCT TCG |
| Del-rpmE-R | AAA AAA AGC GCC CTG CGG CGC TTT TTT TTC TGG CGG TAA CCA TAT GAA TAT CCT CCT TAG |
| Del-rpmE2-F | GTA TTT TAT TGT TAT GTT ATA ACA TAA TTG AGA GGG CGC ATG TAG GCT GGA GCT GCT TCG |
| Del-rpmE2-R | TGT TTT GCA TTG CGA AGT GAG TTT AAC ACC TGC ATT TCA TCA TAT GAA TAT CCT CCT TAG |
| KU109 | GTATAAGGAG GGA ATG ATG GAT ATT CCC GAG TCG ATA AAA ATCGGATCCAGAATTCGTGAT |
| KU110 | ATA AAA AAG GTT ATT GCT TAC TCA CTA TTT TGA GTG GGT T GAGCTCGATCCGTCGACC |
| KU111 | GTATAAGGAG GGA ATG ATG GCT ATT CCC GCT TCG ATA AAA ATCGGATCCAGAATTCGTGAT |
| KU123 | CGC GGATCC TTT TAT CGA AGC GGG AAT ACC CAT CAT TCC |
| KU127 | GGA ATG ATG GGT ATT CCC GCT TCG ATA AAA |
| KU127 | GGA ATG ATG GGT ATT CCC GCT TCG ATA AAA |
| KU135 | GCGGTACC GCCTTACACTGAGGTTCCCCATGAAAAAAGGTATTCACCC |
| KU136 | CCC AAGCTT TTA TTT GCT GCC AGG GAT GC |
