# Supplementary material for: A Translation-Aborting Small Open Reading Frame in the Intergenic Region Promotes Translation of a Mg2+ Transporter in Salmonella Typhimurium
Source: mBio. 2021 Apr 13;12(2):e03376-20. doi: 10.1128/mBio.03376-20 (PMC8092293; doi:10.1128/mBio.03376-20)
Supplement: TEXT S1 [file mBio.03376-20-s0001.docx]

Supplemental Material

**A translation-aborting small open reading frame in the intergenic region promotes translation of a Mg^2+^ transporter in *Salmonella* Typhimurium**

Eunna Choi^a^, Yoontak Han^a^, Shinae Park^b^, Hyojeong Koo^b^, Jung-Shin Lee^b^, Eun-Jin Lee^a,^#

^a^ Department of Life Sciences, School of Life Sciences and Biotechnology, Korea University, Seoul, South Korea

^b^ Department of Molecular Bioscience, College of Biomedical Science, Kangwon National University, Chuncheon, South Korea

# Text S1. Supplemental materials and methods

# Construction of plasmids harboring transcriptional fusions to a promoterless *gfp* gene

Plasmid pGFP-*mgtB* harbors a 1280-nt DNA fragment containing the PhoP-dependent *mgtC* promoter, *mgtC*, *mgtQ*, and first 27 codons of *mgtB* fused transcriptionally to the promoterless *gfp* gene (1). It was constructed as follows: a PCR fragment was generated with primers 1746 and mgtB27BR, and 14028 genomic DNA as a template. The resulting PCR product was digested with EcoRI and BamHI and cloned into plasmid pfpv25 digested with the same enzymes.

pGFP-*mgtQ*_ATG→TAG_-*mgtB* plasmid with the *mgtQ* start codon substituted by TAG stop codon was constructed as follows: a PCR fragment was generated with primers 1746 and mgtB27BR, and EL626 genomic DNA as a template. The resulting PCR product was digested with EcoRI and BamHI and cloned into plasmid pfpv25 digested with the same enzymes.

Derivatives of pGFP-*mgtB* with nucleotides substitution in the *mgtCB* intergenic region (IG) were constructed by cloning PCR fragments generated by two rounds of PCR reactions. For the Stem region 1 substitution in the *mgtCB* intergenic region, a first PCR fragment was generated with primers 1746 and stem A mut-R, and a second fragment was generated with primers stem A mut-F and mgtB27BR, both times using 14028s genomic DNA as a template. A third PCR was performed with primers 1746 and mgtB27BR using the two PCR-generated DNA fragments as templates. The resulting PCR product was digested with EcoRI and BamHI and cloned into plasmid pfpv25 digested with the same enzymes.

For the Stem region 2 substitution in the *mgtCB* intergenic region, a first PCR fragment was generated with primers 1746 and KH386, and a second fragment was generated with primers KH382 and mgtB27BR, both times using 14028s genomic DNA as a template. A third PCR was performed with primers 1746 and mgtB27BR using the two PCR-generated DNA fragments as templates. The resulting PCR product was digested with EcoRI and BamHI and cloned into plasmid pfpv25 digested with the same enzymes.

For the Stem region 3 substitution in the *mgtCB* intergenic region, a first PCR fragment was generated with primers 1746 and KH387, and a second fragment was generated with primers KH383 and mgtB27BR, both times using 14028s genomic DNA as a template. A third PCR was performed with primers 1746 and mgtB27BR using the two PCR-generated DNA fragments as templates. The resulting PCR product was digested with EcoRI and BamHI and cloned into plasmid pfpv25 digested with the same enzymes.

For the Stem region 4 substitution in the *mgtCB* intergenic region, a first PCR fragment was generated with primers 1746 and KH388, and a second fragment was generated with primers KH384 and mgtB27BR, both times using 14028s genomic DNA as a template. A third PCR was performed with primers 1746 and mgtB27BR using the two PCR-generated DNA fragments as templates. The resulting PCR product was digested with EcoRI and BamHI and cloned into plasmid pfpv25 digested with the same enzymes.

For the Stem 1:2 compensatory substitution in the *mgtCB* intergenic region, a first PCR fragment was generated with primers 1746 and Stem A mut-R, and a second fragment was generated with primers Stem A mut-F and mgtB27BR, both times using pGFP-*mgtB* Stem region 2 plasmid DNA as a template. A third PCR was performed with primers 1746 and mgtB27BR using the two PCR-generated DNA fragments as templates. The resulting PCR product was digested with EcoRI and BamHI and cloned into plasmid pfpv25 digested with the same enzymes.

For the Stem 2:3 compensatory substitution in the *mgtCB* intergenic region, a first PCR fragment was generated with primers 1746 and KH392, and a second fragment was generated with primers KH390 and mgtB27BR, both times using 14028s genomic DNA as a template. A third PCR was performed with primers 1746 and mgtB27BR using the two PCR-generated DNA fragments as templates. The resulting PCR product was digested with EcoRI and BamHI and cloned into plasmid pfpv25 digested with the same enzymes.

For the Stem 3:4 compensatory substitution in the *mgtCB* intergenic region, a first PCR fragment was generated with primers 1746 and KH393, and a second fragment was generated with primers KH391 and mgtB27BR, both times using 14028s genomic DNA as a template. A third PCR was performed with primers 1746 and mgtB27BR using the two PCR-generated DNA fragments as templates. The resulting PCR product was digested with EcoRI and BamHI and cloned into plasmid pfpv25 digested with the same enzymes.

For pGFP-*mgtQ*_CCG_-*mgtB* plasmid with the *mgtQ* proline codon (CCC) substituted by a synonymous mutation (CCG), a first PCR fragment was generated with primers 1746 and KH430, and a second fragment was generated with primers KH429 and mgtB27BR, both times using 14028s genomic DNA as a template. A third PCR was performed with primers 1746 and mgtB27BR using the two PCR-generated DNA fragments as templates. The resulting PCR product was digested with EcoRI and BamHI and cloned into plasmid pfpv25 digested with the same enzymes.

For pGFP-*mgtQ*_GGG_-*mgtB* plasmid with the *mgtQ* proline codon (CCC) substituted by glycine codon (GGG), a first PCR fragment was generated with primers 1746 and KH389, and a second fragment was generated with primers KH385 and mgtB27BR, both times using 14028s genomic DNA as a template. A third PCR was performed with primers 1746 and mgtB27BR using the two PCR-generated DNA fragments as templates. The resulting PCR product was digested with EcoRI and BamHI and cloned into plasmid pfpv25 digested with the same enzymes.

For pGFP-*mgtQ*_CTC_-*mgtB* plasmid with the *mgtQ* proline codon (CCC) substituted by leucine codon (CTC), a first PCR fragment was generated with primers 1746 and KH923, and a second fragment was generated with primers KH922 and mgtB27BR, both times using 14028s genomic DNA as a template. A third PCR was performed with primers 1746 and mgtB27BR using the two PCR-generated DNA fragments as templates. The resulting PCR product was digested with EcoRI and BamHI and cloned into plasmid pfpv25 digested with the same enzymes.

For pGFP-*mgtQ*_CAC_-*mgtB* plasmid with the *mgtQ* proline codon (CCC) substituted by histidine codon (CAC), a first PCR fragment was generated with primers 1746 and KH432, and a second fragment was generated with primers KH431 and mgtB27BR, both times using 14028s genomic DNA as a template. A third PCR was performed with primers 1746 and mgtB27BR using the two PCR-generated DNA fragments as templates. The resulting PCR product was digested with EcoRI and BamHI and cloned into plasmid pfpv25 digested with the same enzymes.

For pGFP-*mgtQ*_CGC_-*mgtB* plasmid with the *mgtQ* proline codon (CCC) substituted by arginine codon (CGC), a first PCR fragment was generated with primers 1746 and KH434, and a second fragment was generated with primers KH433 and mgtB27BR, both times using 14028s genomic DNA as a template. A third PCR was performed with primers 1746 and mgtB27BR using the two PCR-generated DNA fragments as templates. The resulting PCR product was digested with EcoRI and BamHI and cloned into plasmid pfpv25 digested with the same enzymes.

p*_lac1-6_*-GFP is a plasmid where the p*_lac1-6_* promoter drives transcription of a promoterless *gfp* gene. It was constructed as follows: a PCR fragment was generated with primers 9903 and 9904, and EG17098 DNA as a template. The resulting PCR product was digested with EcoRI and XbaI and cloned into plasmid pfpv25 digested with the same enzymes.

**Construction of strains with chromosomal mutations in the *mgtQ* ORF**

To generate strains with chromosomal mutations in the *mgtQ* ORF and no additional markers, we implemented the fusaric acid method as described (2). DNA fragments carrying the mutation at the start codon in the *mgtQ* were prepared by a two-step PCR reaction. For the first PCR reaction, we used two primer pairs 7554/10799 and 10798/1259, and 14028 genomic DNA as a template. For the second PCR reaction, we mixed the two PCR products from the first PCR reaction as templates and amplified a DNA fragment using primers 7554 and 1259. The resulting PCR products were purified and integrated into the EG18750 (*mgtCB*::Tet^R^) chromosome harboring pKD46 and selected against Tet^R^ with media containing fusaric acid to generate EL626, a Tet^S^ Amp^S^ chromosomal mutant.

For the Asp2 and Glu5 to Ala substitutions in the *mgtQ*, DNA fragments carrying the Ala substitutions in the *mgtQ* were prepared by a two-step PCR reaction. For the first PCR reaction, we used two primer pairs 7554/KU35 and KU34/1259, and 14028 genomic DNA as a template. For the second PCR reaction, we mixed the two PCR products from the first PCR reaction as templates and amplified a DNA fragment using primers 7554 and 1259. The resulting PCR products were purified and integrated into the EG18750 (*mgtCB*::Tet^R^) chromosome and selected against Tet^R^ with media containing fusaric acid to generate EN1389, a Tet^S^ Amp^S^ chromosomal mutant. The presence of the expected substitution was verified by DNA sequencing.

# Construction of plasmids harboring translational fusions to a promoterless *gfp* gene

Plasmid ptGFP-*mgtB* harbors a 1280 nt-long DNA fragment containing the PhoP-dependent *mgtC* promoter, the *mgtC* gene, *mgtQ*, and first 27 codons of *mgtB* fused in frame to the start codon-less *gfp* gene (1). It was constructed as follows: a PCR fragment was generated with primers 1746 and mgtB27BR, and 14028 genomic DNA as a template. The resulting PCR product was digested with EcoRI and BamHI and cloned into plasmid ptGFP digested with the same enzymes.

ptGFP-*mgtQ*_ATG→TAG_-*mgtB* plasmid with the *mgtQ* start codon substituted by TAG stop codon, was constructed as follows: a PCR fragment was generated with primers 1746 and mgtB27BR, and EL626 genomic DNA as a template. The resulting PCR product was digested with EcoRI and BamHI and cloned into plasmid ptGFP digested with the same enzymes.

Derivatives of ptGFP-*mgtB* with nucleotides substitution in the *mgtCB* intergenic region (IG) were constructed by cloning PCR fragments generated by two rounds of PCR reactions. For the Stem region 1 substitution in the *mgtCB* intergenic region, a first PCR fragment was generated with primers 1746 and stem A mut-R, and a second fragment was generated with primers stem A mut-F and mgtB27BR, both times using 14028s genomic DNA as a template. A third PCR was performed with primers 1746 and mgtB27BR using the two PCR-generated DNA fragments as templates. The resulting PCR product was digested with EcoRI and BamHI and cloned into plasmid ptGFP digested with the same enzymes.

For the Stem region 2 substitution in the *mgtCB* intergenic region, a first PCR fragment was generated with primers 1746 and KH386, and a second fragment was generated with primers KH382 and mgtB27BR, both times using 14028s genomic DNA as a template. A third PCR was performed with primers 1746 and mgtB27BR using the two PCR-generated DNA fragments as templates. The resulting PCR product was digested with EcoRI and BamHI and cloned into plasmid ptGFP digested with the same enzymes.

For the Stem region 3 substitution in the *mgtCB* intergenic region, a first PCR fragment was generated with primers 1746 and KH387, and a second fragment was generated with primers KH383 and mgtB27BR, both times using 14028s genomic DNA as a template. A third PCR was performed with primers 1746 and mgtB27BR using the two PCR-generated DNA fragments as templates. The resulting PCR product was digested with EcoRI and BamHI and cloned into plasmid ptGFP digested with the same enzymes.

For the Stem region 4 substitution in the *mgtCB* intergenic region, a first PCR fragment was generated with primers 1746 and KH388, and a second fragment was generated with primers KH384 and mgtB27BR, both times using 14028s genomic DNA as a template. A third PCR was performed with primers 1746 and mgtB27BR using the two PCR-generated DNA fragments as templates. The resulting PCR product was digested with EcoRI and BamHI and cloned into plasmid ptGFP digested with the same enzymes.

For the Stem 1:2 compensatory substitution in the *mgtCB* intergenic region, a first PCR fragment was generated with primers 1746 and KH389, and a second fragment was generated with primers KH385 and mgtB27BR, both times using ptGFP-*mgtB* Stem region 2 plasmid DNA as a template. A third PCR was performed with primers 1746 and mgtB27BR using the two PCR-generated DNA fragments as templates. The resulting PCR product was digested with EcoRI and BamHI and cloned into plasmid ptGFP digested with the same enzymes.

For the Stem 2:3 compensatory substitution in the *mgtCB* intergenic region, a first PCR fragment was generated with primers 1746 and KH392, and a second fragment was generated with primers KH390 and mgtB27BR, both times using 14028s genomic DNA as a template. A third PCR was performed with primers 1746 and mgtB27BR using the two PCR-generated DNA fragments as templates. The resulting PCR product was digested with EcoRI and BamHI and cloned into plasmid ptGFP digested with the same enzymes.

For the Stem 3:4 compensatory substitution in the *mgtCB* intergenic region, a first PCR fragment was generated with primers 1746 and KH393, and a second fragment was generated with primers KH391 and mgtB27BR, both times using 14028s genomic DNA as a template. A third PCR was performed with primers 1746 and mgtB27BR using the two PCR-generated DNA fragments as templates. The resulting PCR product was digested with EcoRI and BamHI and cloned into plasmid ptGFP digested with the same enzymes.

For ptGFP-*mgtQ*_CCG_-*mgtB* plasmid with the *mgtQ* proline codon (CCC) substituted by a synonymous mutation (CCG_Pro_), a first PCR fragment was generated with primers 1746 and KH430, and a second fragment was generated with primers KH429 and mgtB27BR, both times using 14028s genomic DNA as a template. A third PCR was performed with primers 1746 and mgtB27BR using the two PCR-generated DNA fragments as templates. The resulting PCR product was digested with EcoRI and BamHI and cloned into plasmid ptGFP digested with the same enzymes.

For ptGFP-*mgtQ*_GGG_-*mgtB* plasmid with the *mgtQ* proline codon (CCC) substituted by glycine codon (GGG), a first PCR fragment was generated with primers 1746 and KH389, and a second fragment was generated with primers KH385 and mgtB27BR, both times using 14028s genomic DNA as a template. A third PCR was performed with primers 1746 and mgtB27BR using the two PCR-generated DNA fragments as templates. The resulting PCR product was digested with EcoRI and BamHI and cloned into plasmid ptGFP digested with the same enzymes.

For ptGFP-*mgtQ*_CTC_-*mgtB* plasmid with the *mgtQ* proline codon (CCC) substituted by leucine codon (CTC), a first PCR fragment was generated with primers 1746 and KH923, and a second fragment was generated with primers KH922 and mgtB27BR, both times using 14028s genomic DNA as a template. A third PCR was performed with primers 1746 and mgtB27BR using the two PCR-generated DNA fragments as templates. The resulting PCR product was digested with EcoRI and BamHI and cloned into plasmid ptGFP digested with the same enzymes.

For ptGFP-*mgtQ*_CAC_-*mgtB* plasmid with the *mgtQ* proline codon (CCC) substituted by histidine codon (CAC), a first PCR fragment was generated with primers 1746 and KH432, and a second fragment was generated with primers KH431 and mgtB27BR, both times using 14028s genomic DNA as a template. A third PCR was performed with primers 1746 and mgtB27BR using the two PCR-generated DNA fragments as templates. The resulting PCR product was digested with EcoRI and BamHI and cloned into plasmid ptGFP digested with the same enzymes.

For ptGFP-*mgtQ*_CGC_-*mgtB* plasmid with the *mgtQ* proline codon (CCC) substituted by arginine codon (CGC), a first PCR fragment was generated with primers 1746 and KH434, and a second fragment was generated with primers KH433 and mgtB27BR, both times using 14028s genomic DNA as a template. A third PCR was performed with primers 1746 and mgtB27BR using the two PCR-generated DNA fragments as templates. The resulting PCR product was digested with EcoRI and BamHI and cloned into plasmid ptGFP digested with the same enzymes.

For ptGFP-*mgtQ* (Asp2, Glu5→Ala)*-mgtB* plasmid with the 2^nd^ aspartate codon (GAT) and 5^th^ glutamate codon (GAG) in *mgtQ* substituted by alanine codon (GCT), a first PCR fragment was generated with primers 1746 and KU35, and a second fragment was generated with primers KU34 and mgtB27BR, both times using 14028s genomic DNA as a template. A third PCR was performed with primers 1746 and mgtB27BR using the two PCR-generated DNA fragments as templates. The resulting PCR product was digested with EcoRI and BamHI and cloned into plasmid ptGFP digested with the same enzymes.

For ptGFP-*mgtQ* (Asp2→Gly, Glu5→Ala)*-mgtB* plasmid with the 2^nd^ aspartate codon (GAT) and 5^th^ glutamate codon (GAG) in *mgtQ* substituted by glycine codon (GGT) and alanine codon (GCT) respectively, a first PCR fragment was generated with primers 1746 and KU128, and a second fragment was generated with primers KU127 and mgtB27BR, both times using 14028s genomic DNA as a template. A third PCR was performed with primers 1746 and mgtB27BR using the two PCR-generated DNA fragments as templates. The resulting PCR product was digested with EcoRI and BamHI and cloned into plasmid ptGFP digested with the same enzymes.

Plasmid p*_lac1-6_*-′GFP harbors the p*_lac1-6_* promoter, the ribosome binding sequence, and first two codons of the *mgtC* gene fused in frame to the promoterless *gfp* gene. It was constructed as follows: two oligomers, KH019 and KH020, were annealed to each other and ligated into plasmid ptGFP digested with EcoRI and BamHI. The sequence of the resulting constructs was verified by DNA sequencing.

**Construction of plasmids harboring translational fusions to *mgtQ***

PCR fragments corresponding to nucleotides 2-74 downstream of the *mgtC* gene were amplified with primer KH073, which includes the sequence corresponding to the p*_lac1-6_* promoter and either primer KH074 or KH075 (including a stop codon) using 14028s genomic DNA as a template. For the 2,5Ala substitution in the *mgtQ* ORF, a PCR fragment was generated with primer pairs KH073/KU37 Rev-mgtQ(2,5Ala)-BamHI and 14028s genomic DNA as a template. For the 2Gly, 5Ala substitution in the *mgtQ* ORF, a PCR fragment was generated with primer pairs KH073/KU123 and 14028s genomic DNA as a template. The resulting PCR products were digested with EcoRI and BamHI and cloned into plasmid ptGFP digested with same enzymes. The sequences of resulting constructs were verified by DNA sequencing.

**Construction of strains with C-terminally myc-tagged *mgtQ* genes at its chromosomal locations**

*Salmonella* strains with C-terminal myc-tag fused to the wild-type *mgtQ* or *mgtQ*_2,5→Ala_ genes were generated by the PCR-based tandem epitope tagging system (3). Km^R^ cassettes for the *mgtQ*-8×myc or *mgtQ*_2,5→Ala_-8×myc genes were PCR amplified from plasmid pBOP508 using KU109/KU110 (for *mgtQ*-8×myc) and KU111/KU110 (for *mgtQ*_2,5→Ala_-8×myc) and the resulting PCR products were integrated into the 14028s chromosome to generate EN1465 (*mgtQ*-8×myc::Km^R^) and EN1467 (*mgtQ*_2,5→Ala_-8×myc::Km^R^) respectively. The *mgtQ*-8×myc (EN1469) or *mgtQ*_2,5→Ala_-8×myc (EN1471) strains were generated by removing Km^R^ cassettes from EN1465 or EN1467 using plasmid pCP20 as described (4).

**Construction of strains with chromosomal deletions of the *rpmE1* and *rpmE2* genes**

*Salmonella* strains deleted for the *rpmE1* or *rpmE2* genes were generated by the one-step gene inactivation method (4). Km^R^ cassettes for the *rpmE1* and *rpmE2* genes were PCR amplified from plasmid pKD4 using del-rpmE1-F/del-rpmE1-R (for *rpmE1*) and del-rpmE2-F/del-rpmE2-R (for *rpmE2*) and the resulting PCR products were integrated into the 14028s chromosome to generate DN584 (*rpmE1*::Km^R^) and DN585 (*rpmE2*::Km^R^) respectively. The *rpmE1* (EN1119) or *rpmE2* (EN1120) strains were generated by removing Km^R^ cassettes from DN584 or DN585 using plasmid pCP20 as described (4). A P22 phage lysate grown in strain DN584 was used to transduce EN1120 *Salmonella* selecting for kanamycin resistance to generate an *rpmE1*, *rpmE2*::Km^R^ strain. The *rpmE1* *rpmE2* strain (EN1368) was generated by removing the Km^R^ cassette from the *rpmE1*, *rpmE2*::Km^R^ strain. P22 phages lysates grown in strain DN584 or DN585 were used to transduce EN1389 (*mgtQ*_2,5→Ala_) *Salmonella* and generate EN1408 (*mgtQ*_2,5→Ala_, *rpmE1*), EN1409 (*mgtQ*_2,5→Ala_, *rpmE2*), or EN1414 (*mgtQ*_2,5→Ala_, *rpmE1 rpmE2*) *Salmonella* as described above.

**Construction of a plasmid harboring the *rpmE1* gene**

Plasmid pBAD33-*rpmE1* was constructed as follows: a PCR fragment corresponding to the *rpmE1* gene was generated with primers KU135 and KU136 using 14028s genomic DNA as a template, digested with KpnI and HindIII, and cloned into pBAD33 digested with the same enzymes. The sequence of resulting construct was verified by DNA sequencing.

**Construction of a strain with the p*_lac1-6_*-driven *mgtCBR* operon at its normal chromosomal location**

A *Salmonella* strain with the p*_lac1-6_*-promoter driven transcription of the *mgtCBR* operon was generated by the one-step gene inactivation method (4). Briefly, a Cm^R^ cassette was PCR amplified from plasmid pKD3 using primers 7030 and 7031, and the resulting PCR product was integrated into the 14028s chromosome to generate strain EG17087 (Cm^R^::p*_lac1-6_*-*mgtCBR*). The Cm^R^ cassette was removed using plasmid pCP20 to generate EG17098 (p*_lac1-6_*-*mgtCBR*) *Salmonella*.

**Mouse virulence assays**

Mouse virulence assays were conducted as described (5, 6). Six- to eight-week-old female C3H/HeN mice were inoculated intraperitoneally with ~10^3^ colony-forming units. Mouse survival was followed for 20 days. Mice were killed when reaching the humane endpoint, defined as the loss of more than 20% of body weight, hunched posture, or ruffled coat, to minimize suffering. Virulence assays were conducted twice with similar outcomes, and the data correspond to groups of five mice. All procedures were performed according to approved protocols by the Institutional Animal Care and Use Committee from Kangwon National University.

**Western Blot Analysis**

Cells were grown for 5 h in 35 ml of N-minimal medium containing 0.01 mM Mg^2+^. Cells were normalized by measuring optical density at 600 nm (OD_600_). Crude extracts were prepared in PBS (Phosphate-buffered saline) buffer by sonication and analyzed as described (6). The data are representative of two independent experiments, which gave similar results.

**Measurement of *gfp* expression**

For measuring *gfp* levels of transcriptional fusion, wild-type *Salmonella* harboring pfpv25 derivatives or the vector were used. And measuring *gfp* levels of translational fusion, wild-type *Salmonella* harboring derivatives of ptGFP lacking the RBS and start codon of the *gfp* gene or the vector (1) were used. Cells were grown overnight in N-minimal medium containing 10 mM Mg^2+^ and ampicillin. 1 ml of the overnight culture was washed twice in N-minimal medium without Mg^2+^ and resuspended in 1 ml of the same medium. The resuspended bacteria were inoculated 1:100 volume in 3 ml of N-minimal medium with 10 mM Mg^2+^ or 0.01 mM Mg^2+^ and ampicillin and grown for 5 h at 37°C with shaking. Fluorescence and OD_600_ of the cultures were measured using Synergy H1 reader (BioTek). GFP expression of a given strain was determined by plotting [fluorescence/OD_600_].

**Quantitative real time-polymerase chain reaction (RT-PCR)**

Total RNA was isolated using RNeasy Kit (Qiagen) according to the manufacturer’s instructions. The purified RNA was quantified using a Nanodrop machine (NanoDrop Technologies). cDNA was synthesized using PrimeScript^TM^ RT reagent Kit (TaKaRa). The mRNA levels of the *mgtC*, *mgtB*, and *rrsH* genes were measured by quantification of cDNA using SYBR Green PCR Master Mix (TOYOBO) and appropriate primers (*mgtC*: 7530/7531, *mgtB*: 7763/7764) and monitored using StepOnePlus Real-Time PCR system (Applied Biosystems, Foster City). The mRNA levels of each target genes were calculated using a standard curve of the 14028s genomic DNA with known concentration and data were normalized to the levels of 16S ribosomal RNA amplified with primers 6970 and 6971.

**References**

1. Lee EJ, Groisman EA. 2012. Tandem Attenuators Control Expression of the *Salmonella* *mgtCBR* Virulence Operon. Mol Microbiol 86:212-24.

2. Maloy SR, Nunn WD. 1981. Selection for loss of tetracycline resistance by *Escherichia coli*. J Bacteriol 145:1110-1.

3. Cho BK, Knight EM, Palsson BO. 2006. PCR-based tandem epitope tagging system for Escherichia coli genome engineering. Biotechniques 40:67-72.

4. Datsenko KA, Wanner BL. 2000. One-step inactivation of chromosomal genes in *Escherichia coli* K-12 using PCR products. Proc Natl Acad Sci U S A 97:6640-5.

5. Choi E, Choi S, Nam D, Park S, Han Y, Lee JS, Lee EJ. 2017. Elongation factor P restricts Salmonella's growth by controlling translation of a Mg2+ transporter gene during infection. Sci Rep 7:42098.

6. Choi S, Choi E, Cho YJ, Nam D, Lee J, Lee EJ. 2019. The Salmonella virulence protein MgtC promotes phosphate uptake inside macrophages. Nat Commun 10:3326.
